# Improved properties of hybrid Al-CNTs via h-BNs coated with ag and ni for ball bearings

**DOI:** 10.1038/s41598-024-84249-8

**Published:** 2025-01-08

**Authors:** Waheed S. Barakat, Ahmed Samir, Omayma A. Elkady, Mohamed Abu-Okail, Abdelkarim Y. A. Mohamed, A. EL-Nikhaily, A. M. I. Abu-Oqail

**Affiliations:** 1 Mechanical Department, Faculty of Technology and Education, Suez University, Suez, 43221 Egypt; 2 Powder Technology Department, Manufacturing Technology Institute, Central Metallurgical R & D Institute, Helwan, 11421 Cairo Egypt; 3 Manufacturing Engineering and Production Technology Department, Modern Academy for Engineering and Technology, Cairo, 11571 Egypt; 4 Mechatronics technology department, Higher Technological Institute, Beni-Suef, Egypt; 5 Production Technology Department, Faculty of Technology and Education, Beni-Suef University, Beni-Suef, 62521 Egypt

**Keywords:** Powder metallurgy, AL matrix, Hybrid reinforcement, CNTs, h-BN, Microstructure, Hot compaction, Electroless deposition, High-energy ball milling, Mechanical properties, Composites, Materials science, Ceramics

## Abstract

Ball bearings face numerous challenges under harsh operating conditions of elevated pressure between the balls and other contacting parts of the bearing like drop in tribological properties. To address these challenges, this paper presents the first successful experimental investigation of incorporating an innovative hexagonal boron nitride (h-BN) into Aluminum-Carbon nanotube (Al-0.6 wt% CNTs) nanocomposites. This was achieved using electroless chemical deposition technique to coat the materials with silver (Ag) and nickel (Ni), improving the wettability and dispersion between the matrix and reinforcement. Various h-BN ratios (2, 4, 6, 8 and 10 wt%) are incorporated and consolidated through high-energy ball milling and hot compaction techniques. The produced samples were tested and analyzed physically, mechanically, tribologically, and microstructurally. X-ray diffraction (XRD) and scanning electron microscope (SEM) analyses were used to explore the new morphologies and structures. The study delves into density, hardness, and wear resistance. The optimal h-BN content is determined to be 8 wt%, enhancing wettability and dispersion within the Al-CNTs matrix. Thus, the properties of hardness, compressive strength, wear rate, and COF at 8 wt% of h-BN content were enhanced by 105%, 60%, 74.5%, and 78.5%, respectively, compared to pure Al. This is due to the uniform scattering of h-BN nanoparticles across the entire surface, despite a significant decrease in relative density. In conclusion, the combination of mechanical alloying, electroless deposition, and hot compaction techniques proves to be effective in producing Al-CNTs/h-BN nanocomposites coated with Ag and Ni nanoparticles.

## Introduction

Ball bearings are essential for minimizing friction and wear in rotating machinery, ensuring smooth and efficient operation. They enhance the longevity and reliability of mechanical systems by supporting radial and axial loads with minimal resistance^1^. Used in a wide range of applications, from automotive engines to household appliances, ball bearings significantly improve performance and energy efficiency. Main issues with ball bearings include wear from friction and stress, leading to performance decline and potential failure^2^. Contamination by dirt and dust can cause abrasion, shortening lifespan. Inadequate lubrication raises friction, overheating, and wear. Misalignment causes uneven loads and early failure. Moisture exposure leads to corrosion and material damage. Repeated stress causes fatigue and cracks; overloading results in excessive stress and deformation, reducing lifespan^3^. Ball bearings are made from a variety of materials chosen for strength, durability, and wear resistance. These include high-carbon chromium steel (e.g., AISI 52100) for its hardness and fatigue resistance, stainless steel for strength and corrosion resistance in moisture-prone environments, ceramics like silicon nitride known for high hardness and low density, polymers such as PTFE and nylon offering low friction and chemical resistance, and bronze alloys like phosphor bronze or aluminum bronze for high load capacity and durability in specific industrial applications^4^.

Metal matrix composites (MMCs) represent a highly promising material choice to meet rising demands, featuring tailored properties that are increasingly applied across diverse industries. These composites comprise a base metal matrix and reinforcing inclusions, combining matrix ductility with reinforcement strength to maintain their advantageous characteristics^5,6^. The adoption of MMCs is swiftly expanding in modern applications, mirroring a global surge in demand^7^.

Hybrid metal matrix nanocomposites (HMMNCs) represent an exciting and promising advancement in materials science and engineering^8–10^. These materials are developed by incorporating nanoparticles and/or nanofibers into a metal matrix to create a composite material with enhanced properties. HMMNCs offer several advantages and are considered the next generation of metal matrix nanocomposites (MMNCs) for advanced industrial applications^9,11,12^. Incorporating nanoparticles, such as CNTs, graphene, or ceramic nanoparticles, into a metal matrix can significantly enhance the mechanical properties of the resulting HMMNCs. This includes improved strength, stiffness, hardness, and wear resistance. HMMNCs exhibit enhanced thermal conductivity and stability, making them suitable for applications that involve high-temperature environments, such as aerospace and automotive components. By reducing the weight of the material while maintaining high strength, HMMNCs are beneficial in applications where weight savings are crucial, such as in the aerospace and automotive industries^13–15^. HMMNCs can be engineered to exhibit specific electrical and magnetic properties, making them suitable for applications like electromagnetic shielding or electronic packaging^16^. Certain HMMNCs can offer superior corrosion resistance, which is particularly important in applications where materials are exposed to harsh environments, such as offshore structures^8,17^. The addition of nanoparticles enhances the tribological (friction and wear) properties of HMMNCs, making them suitable for applications like bearings and other mechanical components^18^. HMMNCs have applications in a various industries, including aerospace, automotive, electronics, energy, and more^19^. They are particularly promising for advanced engineering applications where high-performance materials are required. As technology and materials science continue to advance, HMMNCs are likely to play a vital role in meeting the demands of advanced industrial applications by offering materials with improved performance and tailored properties.

Researchers and engineers continue to explore new combinations and manufacturing techniques to unlock the full potential of these innovative materials. Among metal matrices, the Al metal matrix composites (AMMCs) have significant attention in many industrial applications like the aerospace and automotive sectors. AMMCs have emerged as pivotal materials within industrial applications, owing to their distinctive properties that outdo traditional materials^9,11^. The AMMCs amalgamate the lightweight attribute of Al with the superior properties of reinforcements like carbon fibers and ceramics, thereby addressing the pressing need for high-performance materials across many industrial applications^20^. Among these reinforcements, CNTs have garnered considerable attention due to their exceptional properties and capacity to improve AMMCs’ performance substantially^21^. The addition of CNTs significantly enhances the mechanical properties of the AMMCs. The presence of CNTs with the Al-matrix makes a reinforcing network that effectively impedes dislocation movement, augmenting strength and hardness^22^. Beyond mechanical enhancements, CNTs also confer unique thermal and electrical conductivity benefits, giving it to Al-matrix when combined^23^. Therefore, in recent years, many investigations have been conducted to improve the physical, mechanical and tribological properties of AMMCs by incorporating CNTs as reinforcements using different production techniques^24^. For instance, The hot extruded Al-based composites reinforced with 1 wt% CNT was investigated by Kwon and Leparoux^25^. They concluded that the tensile strength of the Al/1 wt% CNT composites improved by around 157% over the produced pure Al due to the uniform distribution and bridging effect. Esawi and El Borady^26^. produced Al-CNT composites with 0.5, 1, and 2% CNT produced by a planetary mill, followed by a rolling process. They noted that the tensile strength was improved by 10% compared to pure Al. Other investigations by Esawi et al.^27^. also exhibited around a 50% improvement in the tensile strength of the Al/2 wt% CNT produced by the extrusion process. As mentioned above, CNTs are a suitable reinforcement for producing Al/CNTs-based hybrid composites. Accordingly, many researchers were interested in producing Al/CNTs-based composites using different reinforcement like Al_2_O_3_^28^, TiO_2_^29^, TiC^30,31^, ZrB_2_^27^, and Gr^32^. Table 1 summarizes the previous works that apply the Al/CNTs as a matrix to produce HMMCs. Another article was focused on improving the efficacy of nanocomposites designed for wind energy applications via coating Al_2_O_3_ with Ag by abu-oqail et al. They also concerned to attain an optimal blend of wear resistance and electrical properties. They examined how graphite exfoliation and silver reinforcement coating impact the composite’s characteristics^33^. Abuoqail et al. reinforced Al using two ceramics: Al_2_O_3_-coated Ni and graphene nanoplatelets (GNPs)-coated Ni through the electroless deposition technique. they used different of contents Al-Al2O3/0, 0.2, 0.6, 1, and 1.4% GNPs hybrid nanocomposite. they concluded that hybrid nanocomposite at 0.6 wt% GNPs exhibited enhanced mechanical and wear properties^34^. The electroless deposition of nanocomposites is extensively utilized in both scientific research and industrial applications to enhance the mechanical and tribological properties of base materials through coating. Recently, electroless nanocomposite coatings have gained popularity in the aerospace, mechanical, chemical, and automotive manufacturing industries due to their ability to create surfaces that are hard, wear-resistant, and corrosion-resistant^35^. Innovative material concepts, especially for coatings, are being successfully applied to optimize critical properties with reduced material consumption, minimal technical effort, and low process costs. In 1844, Wurtz first reported the electroless deposition of metallic nickel from an aqueous solution in the presence of hypophosphite^36^. When a substrate is immersed in an electroless solution, known as an “electro bath,” which contains a metallic ion source, a wetting agent, a complexing agent, a reducing agent, additives, and a stabilizer, it generates a potential. Electroless coatings are classified into four categories: electroless nano coatings, composite coatings, alloy and poly-alloy coatings, and pure Ni and black Ni coatings^37^. The electroless coating method ensures uniform thickness across the entire surface, including edges and intricate inner geometries. Electroless Ni composite coatings are typically created by co-depositing small inert secondary particles into a metallic matrix from an electroless solution to produce metal matrix composites (MMCs). Particle-reinforced metal matrix composites are widely used in engineering applications. Composite electroless deposition is a process that co-deposits metals and particles into a coating. Metals such as nickel, copper, and chromium are commonly used as metal matrices. Hard oxides (Al2O3, ZrO2, and SiO2), carbides (SiC and WC), diamond, and solid lubricants (PTFE, graphite, or MoS2) can be used as co-deposition particles.

Various mechanisms have been developed to enhance the wettability and dispersion of h-BN within the Al-CNT matrix. These include (i) Wettability Enhancement, which involves Surface Energy Modification, Chemical Bonding, and Oxidation Resistance, and (ii) Dispersion Improvement, which encompasses Particle Coating, Interfacial Compatibility, and Electrostatic Stabilization. Specifically, Silver (Ag) and nickel (Ni) coatings enhance the wettability and dispersion of h-BN within the Al-CNT matrix through several mechanisms. For wettability enhancement, these coatings modify the surface energy of h-BN particles, reducing the surface tension between h-BN and the Al-CNT matrix, making it easier for the sintered aluminum to wet and spread over the h-BN surface. They also form interfacial bonds with both h-BN and aluminum, creating a stronger interface that enhances wettability, with Ni forming intermetallic compounds with aluminum for improved adhesion. Additionally, Ag and Ni coatings protect h-BN from oxidation during high-temperature processes, maintaining its structural integrity. For dispersion improvement, coating h-BN particles with Ag or Ni creates a barrier that prevents agglomeration, promoting a more uniform distribution within the Al-CNT matrix. The metallic coatings also enhance interfacial compatibility, reducing the tendency of h-BN particles to cluster together, and in some cases, provide electrostatic stabilization, repelling particles from each other to further improve their dispersion in the matrix. Ramachandran et al.^38^ focused on the fabricated Al₂O₃-MWCNTs composites using spark plasma sintering at 1400 °C, varying the MWCNTs and alumina percentages. They assessed the composites’ physical, mechanical, and thermal properties, confirming phases of α-Al₂O₃ and carbon using techniques like SEM, XRD, and elemental analysis. Incorporating 1 wt% MWCNTs into Al₂O₃ significantly enhanced hardness (17.26 ± 0.4 GPa), fracture toughness (K_IC = 5.6 ± 0.3 MPa m^1/2), and flexural strength (515 ± 33 MPa). Moreover, Arunkumar et al.^39^ developed the hybrid cermet’s using Al 6061 alloy reinforced with nano-sized SiC, Al₂O₃, and TiO₂ through a novel ultrasonic rheo-squeeze casting technique. This process, involving ultrasonication at 20 kHz and 50 MPa pressure, improved the physical, thermal, and mechanical properties of the cermet’s, especially those with Al₂O₃ reinforcements. The resulting cermet’s demonstrated enhanced mechanical performance, reduced cavities, and increased resistance to corrosion and abrasive wear by approximately 97% and 71%, respectively. Arunkumar et al.^40^ investigated the mass production of multi-walled carbon nanotubes (MWCNTs) via thermal catalytic chemical vapor deposition (TC-CVD). MWCNT synthesis involved the breakdown of acetylene (C2H2) gas with Fe/MgO catalysts. The surface morphology and structure of the MWCNTs, confirmed through scanning electron microscopy (SEM), revealed particle sizes of 20–30 nm. XRD determined phase identification and crystalline size, while EDAX conducted elemental analysis. Additionally, thermogravimetric analysis (TGA) was used to study the material’s thermal properties. Arunkumar et al.^41^ studied the effect of strengthened YSZ nano-ceramics with TC-CVD-synthesized MWCNTs through spark plasma sintering at 1350 °C. XRD, SEM, and EDS were employed to analyze phase changes, microstructure, and elemental composition. The research explored physical and mechanical traits, encompassing density, porosity, hardness, fracture toughness, and wear resistance. Despite reduced hardness at higher MWCNT concentrations due to agglomeration, wear resistance improved due to mechanisms like pull-out and crack branching. Fracture toughness assessments using indentation and single-edge V-notch beam methods revealed that YC1 exhibited 21% higher toughness (6.58 ± 0.3 MPa m^1/2) than YSZ (5.21 ± 0.2 MPa m^1/2), attributed to MWCNTs’ reinforcing effects such as crack deflection and bridging. Based on prior research, the Al/CNTs matrix was utilized, with CNT concentrations ranging from 0.5 to 1 wt%, to create HMMCs through the powder metallurgy (PM) technique. Previous studies have explored various methods to enhance composites’ wear properties. Some employed techniques involve coating material surfaces with liquid or solid lubricants. However, solid lubricant coatings have drawbacks such as oxidation, weak bonding, and a limited lifespan^9,34^, while liquid lubricants prove unsuitable under challenging weather conditions^19,35,36^. To address these challenges, integrating solid lubricants into the metal matrix offers an alternative approach to reduce wear and friction without relying on liquid lubricants^37^. h-BN, when incorporated into Metal Matrix Composites (MMCs), emerges as a promising reinforcement material. The advantages of adding h-BN to MMCs include its self-lubricating properties and low coefficient of friction. The self-lubrication of h-BN is particularly advantageous in applications were traditional liquid lubricants face limitations, such as in high-temperature environments or vacuum conditions^15,38–40^.

**Table 1:** The previous works related to applying the Al/CNTs as a matrix to produce HMMCs.

No	Al-CNTs	Reinforcement	Production technique	Ref.
1.	Al-0.5 wt% CNTs	ZrB_2_ 5 wt%	Powder metallurgy	^27^
2.	Al-1 vol% CNTs	TiO_2_ 0–6 vol%		^29^
3.	Al-0.6 wt% CNTs	TiC 10 wt%		^31^
4.		0–2 wt%	Stir casting	^30^
5.	Al-2 wt% CNTs	Gr 0–5 wt%		^32^
6.		Al_2_O_3_ 2 wt%	Accumulative roll bonding	^28^

According to the literature survey, it is evident that researchers have not investigated the impact of adding h-BN to create Al/CNTs/h-BN hybrid composites, combining the advantages of CNTs^13,21–24,33,41–43^. and h-BN^15,39,40,44–48^ in the Al-matrix. This combination makes the material well-suited for automotive and industrial applications. Therefore, this study aims to explore the synergistic effects of nano-sized CNTs and h-BN as reinforcement materials on the microstructure, densification, hardness, compressive strength, and wear performance of Al/0.6 wt%/ (0–10 wt%) h-BN composites produced through the powder metallurgy technique. It’s worth noting that the surfaces of CNTs and h-BN were modified by coating them with Ni using electroless deposition to enhance the wettability between the Al-matrix and the reinforcements.

This study is motivated by the imperative to overcome limitations and enhance the performance of metal matrix composites (MMCs), specifically targeting improvements in wear resistance and friction reduction. Previous research has explored various methods to enhance wear properties of composites, including coatings with liquid or solid lubricants. However, these approaches suffer from issues such as oxidation, weak bonding, and limited durability. Moreover, traditional lubricants are often inadequate under extreme conditions like high temperatures or vacuum environments. To address these challenges, integrating solid lubricants directly into the metal matrix presents a compelling alternative. Hexagonal boron nitride (h-BN) stands out for its self-lubricating properties and low coefficient of friction, offering promise in applications where conventional lubricants fall short.

The novelty of this study lies in several key aspects. Firstly, the combination of h-BN with carbon nanotubes (CNTs) within Al-based composites (Al/CNTs/h-BN hybrid composites) represents a novel approach that has not been extensively explored in existing literature. The study aims to investigate the synergistic effects of nano-sized CNTs and h-BN as reinforcement materials in the Al-matrix, specifically examining the influence of h-BN additions ranging from 0 to 10 wt% on microstructure, densification, hardness, compressive strength, and wear performance of the composites. Furthermore, the study innovates methodologically by employing electroless deposition of Ni to modify the surfaces of CNTs and h-BN. This surface modification aims to enhance the wettability between the Al-matrix and the reinforcements, potentially improving overall composite properties. The practical implications of this research extend to automotive and industrial sectors, where enhanced wear resistance and reduced friction could lead to significant performance benefits. In summary, this study not only addresses current challenges in composite materials but also introduces a novel composite formulation and processing approach to achieve superior performance characteristics in demanding applications.

## Experimental works

**Fig. 1:**
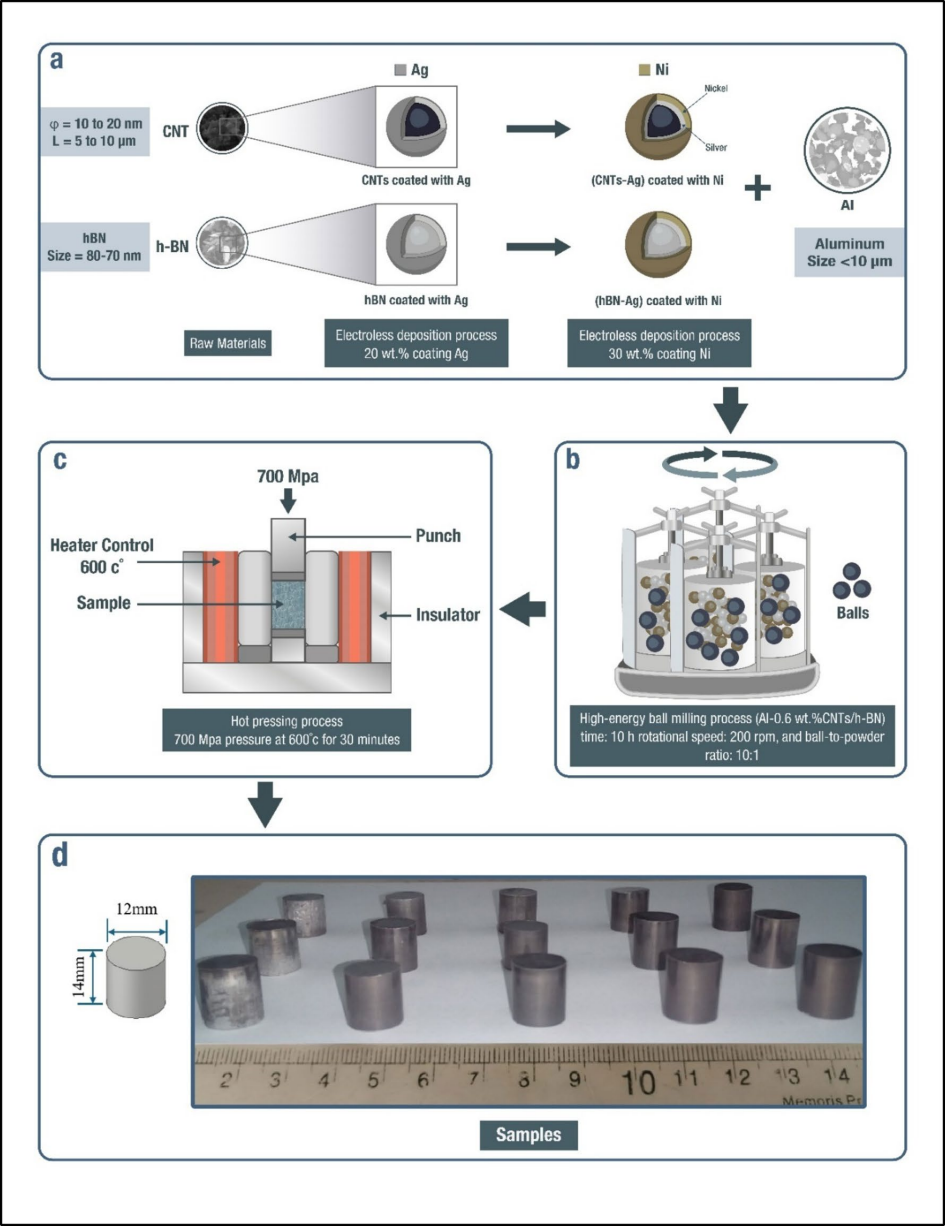
Schematic representation illustrating the production process of Al-0.6% CNTs/h-BN hybrid nano composites with Ag/Ni coating. The process involves three main steps: (**a**) Materials preparation, (**b**) High-energy ball milling process, (**c**) Synthesis of Al-0.6% wt. CNTs/h-BN hybrid nano composites using the hot-pressing process, and (**d**) sintered samples.

This work used the Al-0.6 wt% CNT as a matrix to produce the Al-0.6 wt% CNT hybrid composites reinforced with different concentrations of 0, 2, 4, 6, 8 and 10 wt% h-BN particles. (Fig. 1) illustrates the applied experimental procedures to prepare Al-0.6 wt% CNT/h-BN hybrid composites using PM technique.

### Initial materials

The Al powder purity of 99.99% and particle size < 10 μm were selected as a matrix. It is supplied from (Dop Organic Kimya, Ankara, Turkey). Two ceramic powders reinforced the Al matrix, CNTs, and h-BN to produce Al/CNTs/h-BN nano composites. According to a previous investigation^49^, CNTs were synthesized using an electric arc discharge process, resulting in a purity of over 90%, with diameters ranging from 10 to 20 nm and lengths ranging from 5 to 10 μm. In addition, the nano shell company (City, Country) supplied the h-BN with a purity of 99% and an average particle size of 70–80 nm. The CNTs were added to the matrix with a fixed concentration of 0.6 wt%, and the h-BN particles were added with different concentrations of 0, 2, 4, 6, 8 and 10 wt% to prepare the Al/CNTs/h-BN hybrid nano composites.

**Table 2:** The concentrations of the prepared Al-0.6 wt% CNTs/h-BN composite samples.

	Matrix (wt%)	Reinforcement (wt%)
1	Al pure	0% h-BN
2	97.4% Al – 0.6% CNTs	2% h-BN
3	95.4% Al – 0.6% CNTs	4% h-BN
4	93.4% Al – 0.6% CNTs	6% h-BN
5	91.4% Al – 0.6% CNTs	8% h-BN
6	89.4% Al – 0.6% CNTs	10% h-BN

### Surface modification of CNTs and h-BN

As both CNTS and h-BN are ceramic materials, so there is a non-wettability problem between each one of them with the Al matrix. This due to the high surface energy between them is causing the formation of pores and internal voids in the final product so the surface particles of CNTs and h-BN were coated with Ni nano using the electroless process to improve the bonding between the matrix and the reinforcements. Before the coating of Ni on the surface of particles, pretreatment of CNTs and h-BN by coating Ag for favourable conditions for plating was applied. The electroless nano Ag coating process was employed to deposit nano Ag on the surfaces of either h-BN or CNTs through two primary steps: sensitization and nano Ag deposition. To enhance the surface sensitivity of h-BN and CNTs and effectively remove any surface contamination, they are immersed in a 10% sodium hydroxide solution with continuous stirring for a duration of 1 h, followed by immersion in acetone for an additional hour. Subsequently, the h-BN and CNTs underwent filtration and were subjected to a washing process using distilled water. Following this, the h-BN and CNTs were dried in an electric furnace at a temperature of 110 °C for a duration of 1 h. The electroless Ag deposition at a weight% of 20% on h-BN and CNTs was achieved using a chemical bath. The bath consisted of a solution containing 3 g/L of silver nitrate and 300 ml/L of formaldehyde, and the pH was adjusted to 12 using ammonia. Immediately following the addition of formaldehyde, the reaction commenced. The suspension of h-BN and CNTs in the solution was achieved by subjecting the mixture to magnetic stirring for a duration of 10 min at ambient temperature. The electroless reaction was successfully carried out within a duration of 30 min. The solution underwent filtration and subsequent washing with acetone, followed by vacuum drying at a temperature of 110 °C for a duration of 1 h^12^. The electroless chemical deposition technique is utilized to coat either h-BN or CNTs particles with 30 wt% Ni. To carry out this process, several steps are followed: Initially, the required amounts of nickel chloride, Potassium sodium tartrate, ammonium chloride, and Sodium hypophosphite are calculated as 100 g/L, 80 g/L, 50 g/L, and 100 g/L, respectively. The pH is adjusted to approximately 9.2, and the temperature is set to around 92 degrees Celsius. Finally, ceramic powders are added separately to the mixture^16^.

### Fabrication of Al-CNTs/h-BN hybrid nano composites

The hybrid nanocomposite of Al-CNTs/h-BN was fabricated using the following procedure. The samples were combined in the previously specified concentrations (Table 2) and subsequently subjected to high-energy ball milling using a planetary milling machine for 10 h.; with a rotational speed of 200 rpm. The ball-to-powder ratio was 10:1, with the ball diameter ranging from 10 to 12 mm. The hot-pressing process for the milled samples was conducted at 600 °C (increased gradually at 10 ºC/min) for 30 min. and conducted under a pressure of 700 MPa (Fig. 1). The sample should be pressed into a cylindrical shape with a diameter of 12 mm. The experiments were conducted multiple times to ensure the reproducibility of the results.

### Characterizations of Al-CNTs/h-BN hybrid nano composites

The microstructures of the Initial material (before and after the electroless process) and the fabricated Al-CNTs/h-BN hybrid composites were studied using a QUANTAFEG250 scanning electron microscope (SEM) equipped with energy dispersive X-ray (EDS) spectrometers utilizing the backscattered electron and secondary electron detection modes. The technique employed in this study allowed for the evaluation of the powder particle size, morphology, and microstructure features. The initial step in preparing the hybrid composite samples for microstructure observation entailed milling them with fine sandpaper and then polishing them with alumina paste. The phase structure was investigated using X-ray diffraction (XRD) analysis (Device name, model, City, Country), employing Cu k radiation (λ = 0.15406 nm) to assess the potential formation of new phases during the electroless deposition and hot-pressing processes. The density of the sintered hybrid composite samples was assessed according to MPIF standard 42, 1998, employing the Archimedes principle. The samples were measured in both distilled water and air prior to the calculation of their density. The mechanical properties of the obtained composite specimens were evaluated through compressive strength and Vickers microhardness testing. Compression tests, conducted using a uniaxial SHIMADZU universal testing machine (UH-F500KN) in accordance with ASTM E9-89a, provide critical information on the strength and deformation characteristics of composite materials, aiding in their characterization, comparison, and quality assurance. For these tests, cylindrical samples with a height and diameter of 10 mm each (aspect ratio l/d = 1) were prepared and tested at a cross-head speed of 1 mm/min at room temperature. The Vickers hardness device (FM-ARS9000, USA) was used to test the polished hybrid composite samples’ hardness. The test was conducted with a Vickers indenter under a load of 300 g for a dwell time of 15 s. An average of five indentations was used for each sample, following ASTM standard E 92. The wear properties of the prepared composite samples were evaluated using a dry wear test through pin-on-disc, in accordance with ASTM (G77-98). The composite samples (φ12 mm × 12 mm) were used as pins and loaded perpendicularly onto a hardened steel disc with a hardness of 60 HRC and a surface roughness of 60 μm. The wear tests were conducted at room temperature under dry sliding conditions, with the samples subjected to four different normal loads of 5, 10, 15, and 20 N. The sliding speed was kept constant at 1.5 m/s, and the sliding distance was set at 150 m for each test. The rotational speed was fixed at 450 rpm, and no lubrication was applied during the tests. The values of the volumetric wear rate were calculated based on the weight loss of the sample, the density of the material, and the total sliding distance. represented by the Eq. (1)^50^

1 $$\:{V}_{wear}\frac{\varDelta\:m}{\rho\:\cdot\:L},$$

where:

The volumetric wear rate (V wear) is determined by the weight loss (Δm) of the sample divided by the product of the material density (ρ) and the total sliding distance (L), measured in (mm^3^/m).

The worn surfaces selected for the SEM analysis of worn surfaces were examined using the Abbott–Firestone analysis method, both prior to and following sliding wear tests. Warn Surfaces topography data was obtained and processed using GWYDDION software, after which it was transferred to Excel 365 for further analysis. The resulting data was utilized to generate Abbott-Firestone curves, which provide valuable insights into surface properties. The Abbott-Firestone curve was segmented into three distinct functional zones: The peaks area is the uppermost portion of the surface profile, and the exploitation zone is the middle region where most surface interactions occur, and the voids area is the deepest valleys or depressions in the surface.

## Results and discussions

### Characterization of the initial materials

**Fig. 2:**
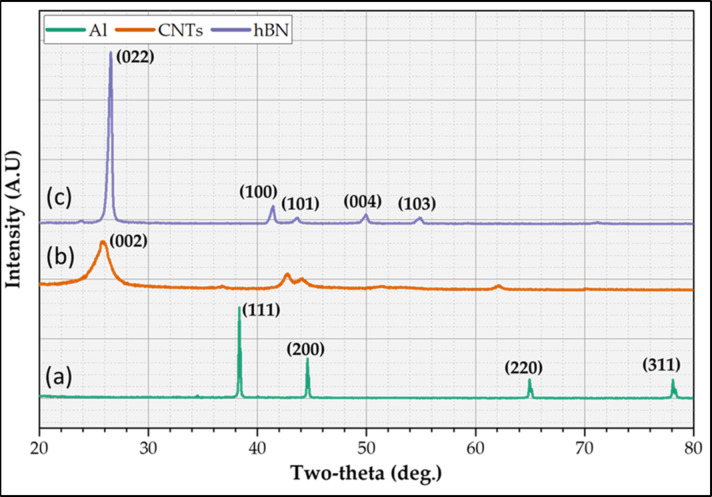
XRD patterns for the initial materials of Al, CNTs, and h-BN.

**Fig. 3:**
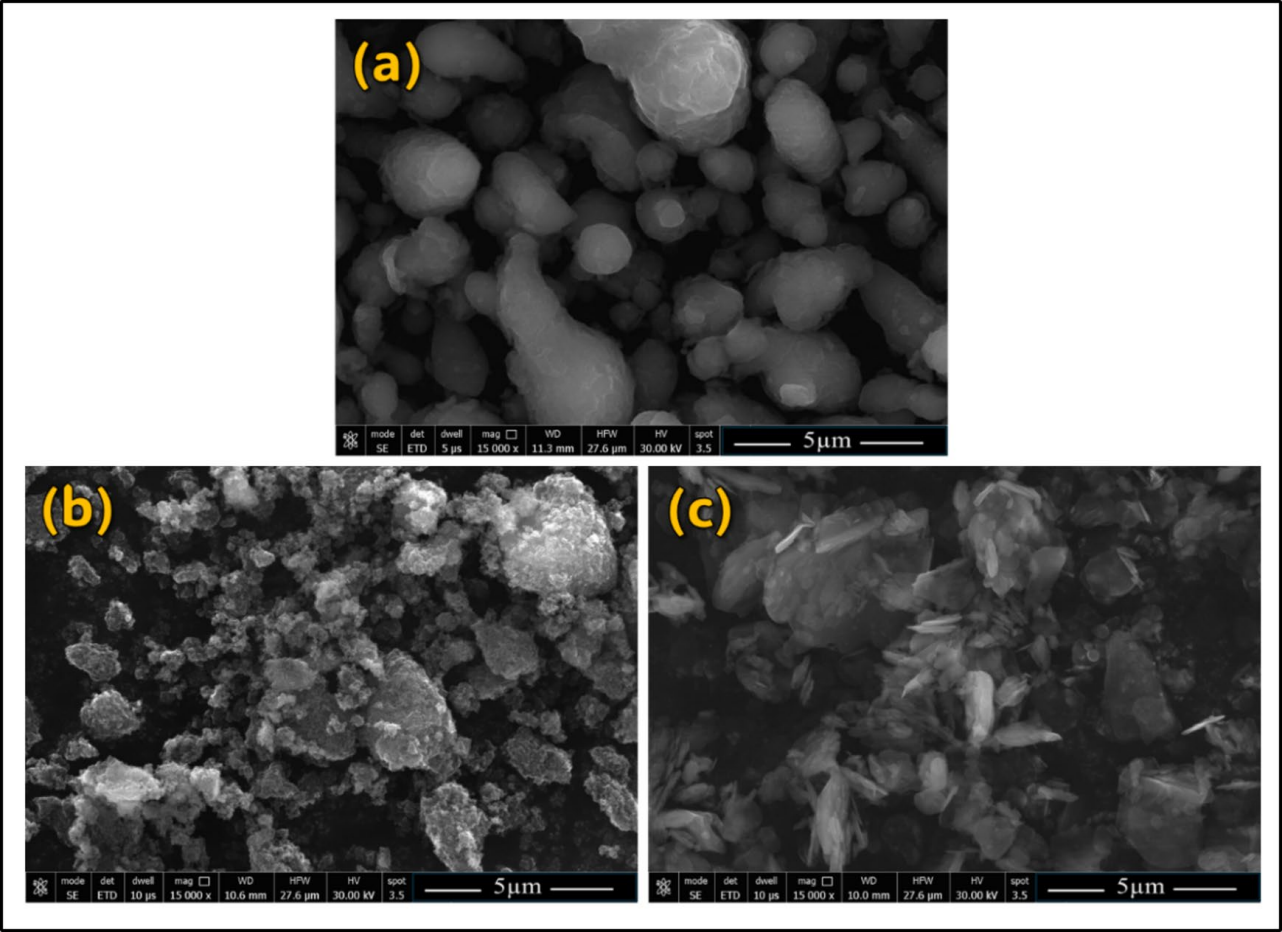
The SEM images illustrate the morphology and structure of each component prior to producing the Al-0.6% CNTs/h-BN Hybrid Nano Composites, The images show (**a**) Al, (**b**) CNTs, and (**c**) h-BN.

The SEM and XRD analyses were applied to detect the morphology and size of the particles and confirm the phase composition and phase structure of the used initial materials by analyzing the diffraction patterns produced. Figure 2 shows the XRD patterns of Al-matrix (Fig. 2a), CNTs (Fig. 2b), and h-BN (Fig. 2c). For the XRD pattern of Al-matrix, It can be shown that the XRD shows a series of peaks corresponding to the (hkl) planes of the FCC lattice, and the prominent peaks of pure Al have planes of (111), (200), (220), and (311), which appeared at around 38.7°, 44.5°, 65.0°, and 78.4° 2-Theta, respectively, as shown (Fig. 2a). The XRD of CNTs reinforcement is confirmed with the high purity CNTs and displays diffraction peaks at 2-Theta values of approximately 25.7° representing the (002), as shown in (Fig. 2b). The sharp peak indexed with (002) shows that the CNTs graphite structure^13,22,25,39,51^. Furthermore, the h-BN XRD pattern (Fig. 2c) exhibits prominent peaks at approximately 27.5°, 41.6°, 43.6° 50.3°, and 54.8° corresponding to the (002), (100), (110), (004), and (103) planes, respectively^12,15,38,40^. It can be concluded that all typical Al (Fig. 2a), CNTs (Fig. 2b), and h-BN (Fig. 2c) and no foreign peaks like oxides and any new phases are remarked, which confirmed the high purity of used initial materials of matrix and reinforcements. Figure 3 depicts the SEM images of the (Fig. 3a) Al-matrix and the reinforcement of (Fig. 3b) CNTs and (Fig. 3c) h-BN. The SEM images confirms the sizes of the Al-matrix and the corresponding reinforcements according to the supplier. Furthermore, the Al particles, CNTs, and nano h-BN have an almost spherical shape, agglomerated in spherical shape (in micron size), and irregular shape with sharp edges, respectively.

**Fig. 4:**
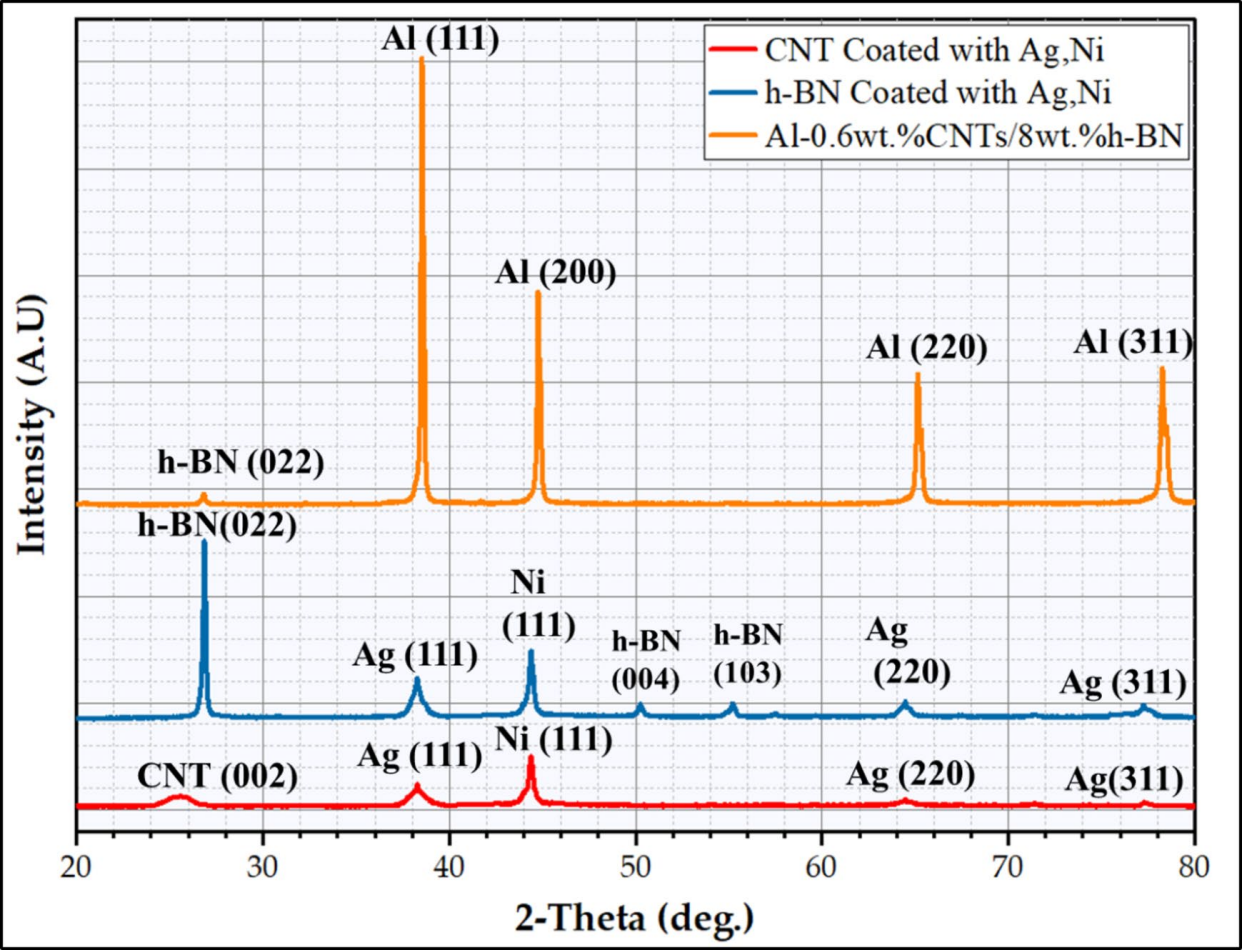
The XRD analysis of the coated CNTs and h-BN with Ag/Ni and the XRD of Al-0.6 wt% CNTs/8 wt% h-BN hybrid nanocomposites.

**Fig. 5:**
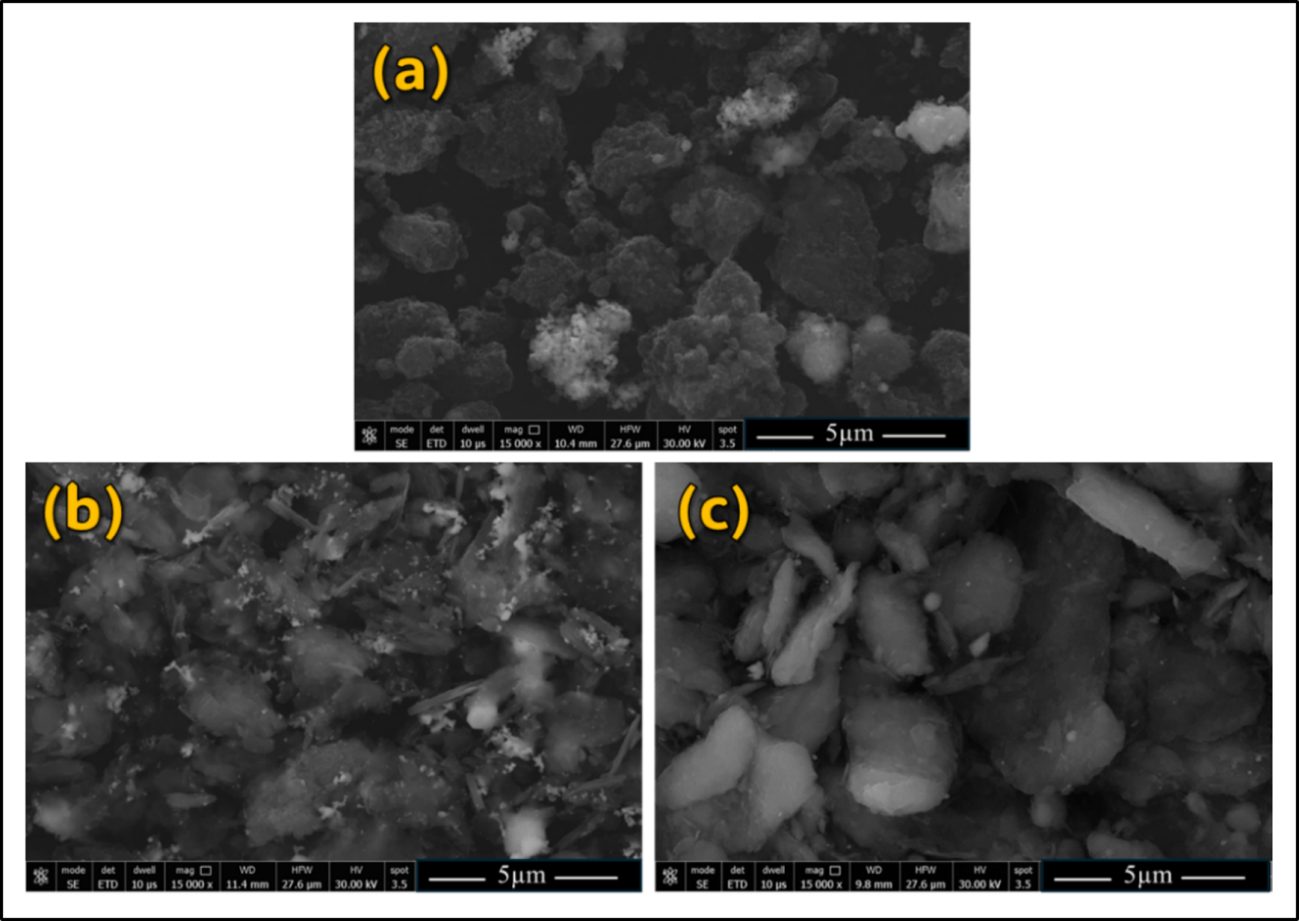
The SEM images of the coated (**a**) CNTs and (**b**) h-BN with Ag/Ni and (**c**) the coated reinforcements of CNTs and h-BN after mixing with Al-matrix.

Figure 4 illustrates the XRD patterns of the CNTs and h-BN reinforcements before and after the coating process with Ag/Ni and the mixed Al-0.6 wt% CNT reinforced with 10 wt% h-BN. The XRD patterns display only the prominent peaks of the used materials of the Al-matrix and their coated reinforcements (CNTs/Ag/Ni and h-BN/Ag/Ni), which also ensures the validity of the electroless coating and mixing processes without contamination of other undesired materials. It can be mentioned that the CNTs, Ag, and Ni peaks were not detected visually due to their low-volume fractions. (Fig. 5) indicates the SEM images of the nano reinforcement of (Fig. 5a) CNTs and h-BN (Fig. 5b) after the coating process with Ag, followed by Ni. It can be remarked that the as-supplied nano reinforcements retained the initial size and shape after the coating process with the presence of dispersed Ag/Ni in the nano spherical shape, as shown in Fig. 5(a,b). The presence of Ni and Ag coated the applied reinforcements is confirmed with the XRD results (Fig. 4). The XRD results and SEM images indicate the success of the electroless chemical deposition technique in depositing the nano-Ag and nano-Ni on the surface of the reinforcements. After the mixing process, (Fig. 5c) shows the SEM image of the mixed Al-0.6 wt% CNTs/8 wt% h-BN nanohybrid composites. It can be revealed that all the particles of the matrix and their reinforcement in the sample are mixed with nearly the shapes and sizes of the as-received powders, and well distributions with no agglomerations were observed, which improves the compressibility of mixed powder during the hot compaction process^12,33,34^.

### Phase analysis and microstructures of sintered nano composites

**Fig. 6:**
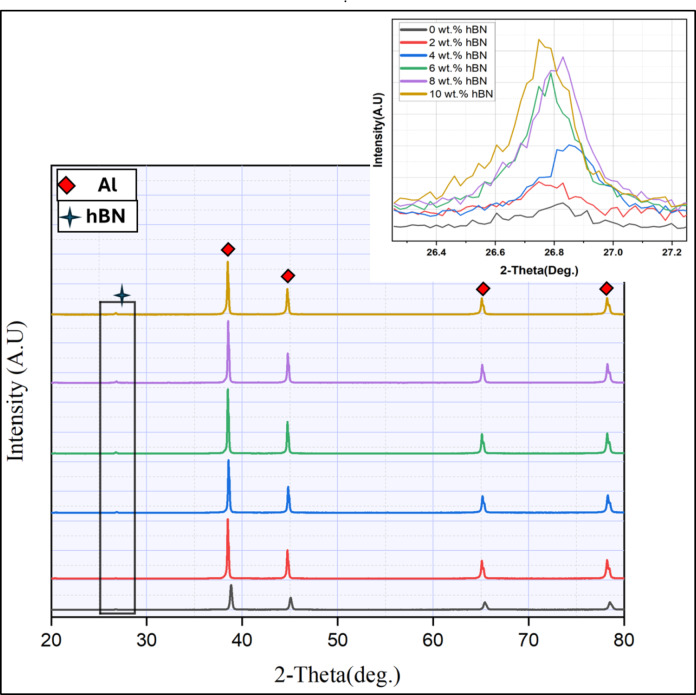
The XRD analysis of the sintered Al-0.6 wt% CNTs/h-BN nanohybrid composites reinforced with the 0, 2, 4, 6, 8 and 10 wt% of nano h-BN.

In Fig. 6, X-ray diffraction (XRD) analysis is showcased for an Al–0.6wt.% CNTs nanocomposite, revealing varying concentrations of h-BN coated Ag and Ni nanoparticles. The fig affirms the components of the produced nanocomposite, indicating the absence of undesired chemical precipitation during the deposition of Ag and Ni particles. The detected peaks corresponding to Al and h-BN demonstrate the controlled sintering parameters, as no undesirable new phases like Al_2_O_3_ or Al_4_C_3_ are observed. Additionally, an increase in the integrity of h-BN peaks is noted with a higher percentage. The crystallite size is generally reduced with increased in coated h-BN content, except for 10wt.% h-BN.

**Fig. 7:**
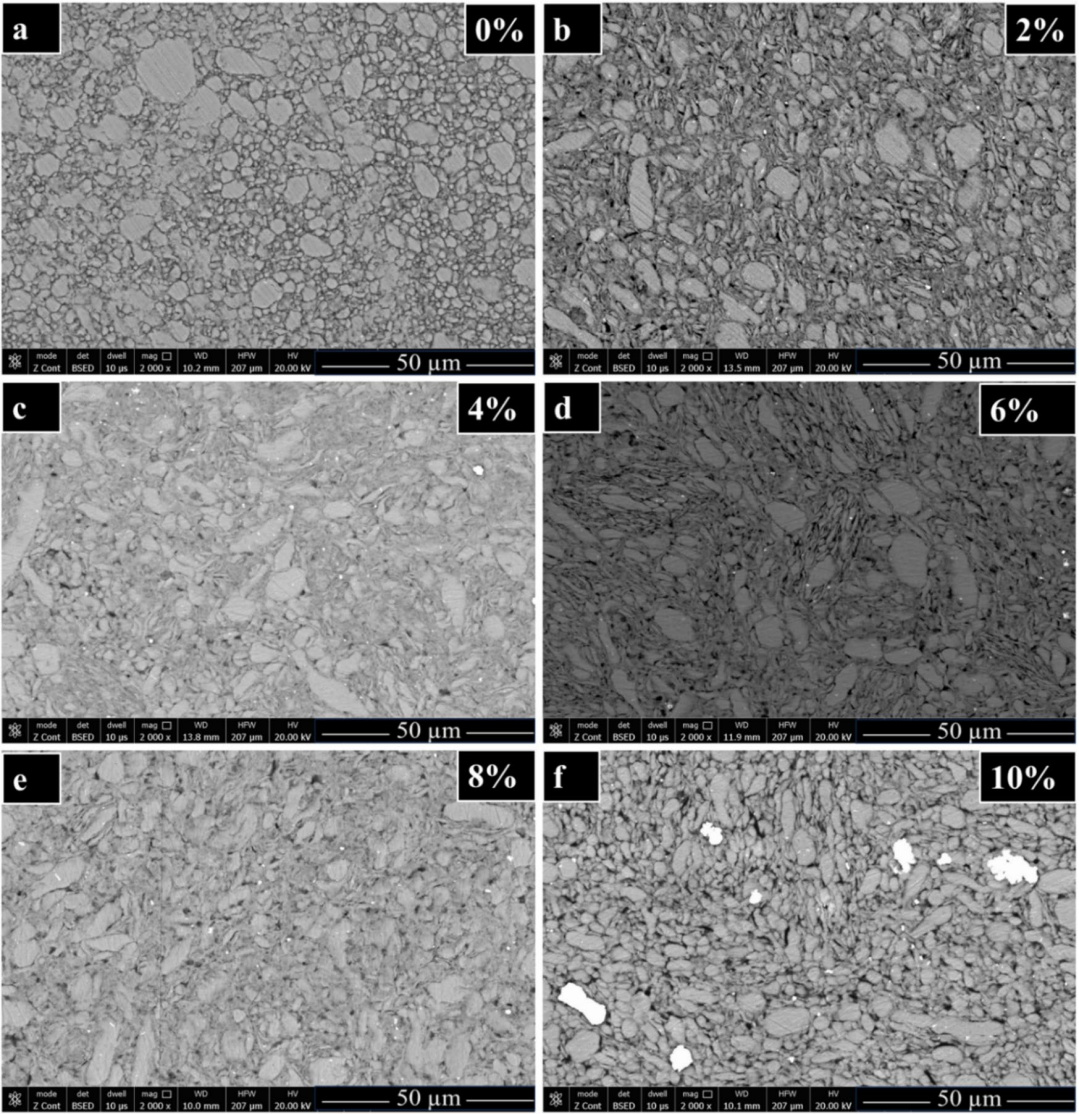
Figure presents the SEM images of the sintered Al-0.6 wt% CNTs/h-BN nanohybrid composite samples with varying amounts of h-BN reinforcement. The images show the microstructural features for (**a**) 0 wt%, (**b**) 2 wt%, (**c**) 4 wt%, (**d**) 6 wt%, (**e**) 8 wt%, and (**f**) 10 wt% of nano h-BN content, respectively.

Figure 7 shows the microstructures of the consolidated samples at various h-BN contents. In Fig. 7(a), we observe the presence of Al grains distributed in an irregular manner. Specifically, the grain size on the left side of the surface is relatively large compared to the size of the grains on the right side of the same surface. Furthermore, it is evident that the distribution of grains exhibits inconsistency across the surface. As for Fig. 7(b), when h-BN was added at a content of 2%, the microstructure improved slightly, as we elucidate that the distribution of the particles became almost semi uniform, and the size of the particles also became smaller than the unreinforced sample with the addition of h-BN. As we can see, the shape of the grains was relatively elongated. It is worth noting that the addition of h-BN helped to coordinate and arrange the grains and was an obstacle to increasing the size of the grains. An important point can be observed that the grains of Ag and Ni are distributed between the Al and h-BN grains, but in an irregular manner. In Fig. 7(c), with an increase in h-BN at 4%, we notice that the Al particles were distributed more regularly than at 2% h-BN, where the particle size decreases relatively. We also notice the appearance of Ag and Ni grains distributed between the Al and CNTs nanoparticles. It is worth noting that some pores appeared densely on the surface, although they were not present in the percentage that contained 2% h-BN. In Fig. 7(d), with an increase in the percentage of h-BN by 6% h-BN, we notice a reduction and decrease in the size of the grains and their distribution, and more regularity in their distribution than it was at the percentage of 4% h-BN. We also notice that at the percentage of 6% h-BN, we notice the best plastic formation and dynamic recrystallization that is clearly visible. This observation is the result of two main factors: (1) h-BN helped the granules slide over each other, reducing the presence of small pores. (2) Adding Ag and Ni coating reduces the phenomenon of wetness, which increases the adhesion of the granules to each other and reduces the internal pores. In Fig. 7(e), we notice that by increasing the percentage of h-BN at 8% h-BN, there is an increase in the plastic formation and dynamic recrystallization, which causes small grains as well as regularity in the particles. Notably, we notice regularity in the distribution of nickel and silver grains between and on the aluminum and carbon nanotube and h-BN, which helped to reduce the size and pores very effectively. This enhancement aims to optimize thermal management in electronics, mechanical components, electrical insulation and conductivity, abrasion and wear resistance, biomedical applications, high-performance composites, and environmental and chemical resistance. Overall, this nanocomposite enhances performance, durability, and efficiency across diverse industrial sectors. In Fig. 7(f), with an increase in the percentage of h-BN at 10%, we notice that the grain size is less than it was at 8%, due to plastic formation and new dynamic recrystallization. Various mechanisms have been observed to enhance the wettability and dispersion of h-BN within the Al-CNT matrix. These include (i) Wettability Enhancement, which involves Surface Energy Modification, Chemical Bonding, and Oxidation Resistance, and (ii) Dispersion Improvement, which encompasses Particle Coating, Interfacial Compatibility, and Electrostatic Stabilization. Specifically, Silver (Ag) and nickel (Ni) coatings enhance the wettability and dispersion of h-BN within the Al-CNT matrix through several mechanisms. For wettability enhancement, these coatings modify the surface energy of h-BN particles, reducing the surface tension between h-BN and the Al-CNT matrix, making it easier for the sintered aluminum to wet and spread over the h-BN surface. They also form interfacial bonds with both h-BN and aluminum, creating a stronger interface that enhances wettability, with Ni forming intermetallic compounds with aluminum for improved adhesion. Additionally, Ag and Ni coatings protect h-BN from oxidation during high-temperature processes, maintaining its structural integrity. For dispersion improvement, coating h-BN particles with Ag or Ni creates a barrier that prevents agglomeration, promoting a more uniform distribution within the Al-CNT matrix. The metallic coatings also enhance interfacial compatibility, reducing the tendency of h-BN particles to cluster together, and in some cases, provide electrostatic stabilization, repelling particles from each other to further improve their dispersion in the matrix. An important point can be observed that, During the mechanical alloying and hot compaction processes, the increase in hexagonal boron nitride (h-BN) content can result in micropore formation due to several contributing factors. These include the agglomeration of h-BN particles, which may not fully break down into finer particles, leading to regions of reduced density in the final compacted material. Additionally, inadequate dispersion of h-BN within the matrix material during ball milling can create uneven distribution, resulting in voids or micropores under pressure during compaction. Oxidation or contamination of h-BN particles or the matrix material during milling can act as nucleation sites for porosity during subsequent compaction. Moreover, residual lubricants from ball milling, if not entirely removed, can decompose during hot compaction, forming voids, while thermal decomposition processes during compaction can also lead to gas evolution and micropore formation. These factors collectively contribute to non-uniform density and the development of micropores in the final ceramic composite material.

**Fig. 8:**
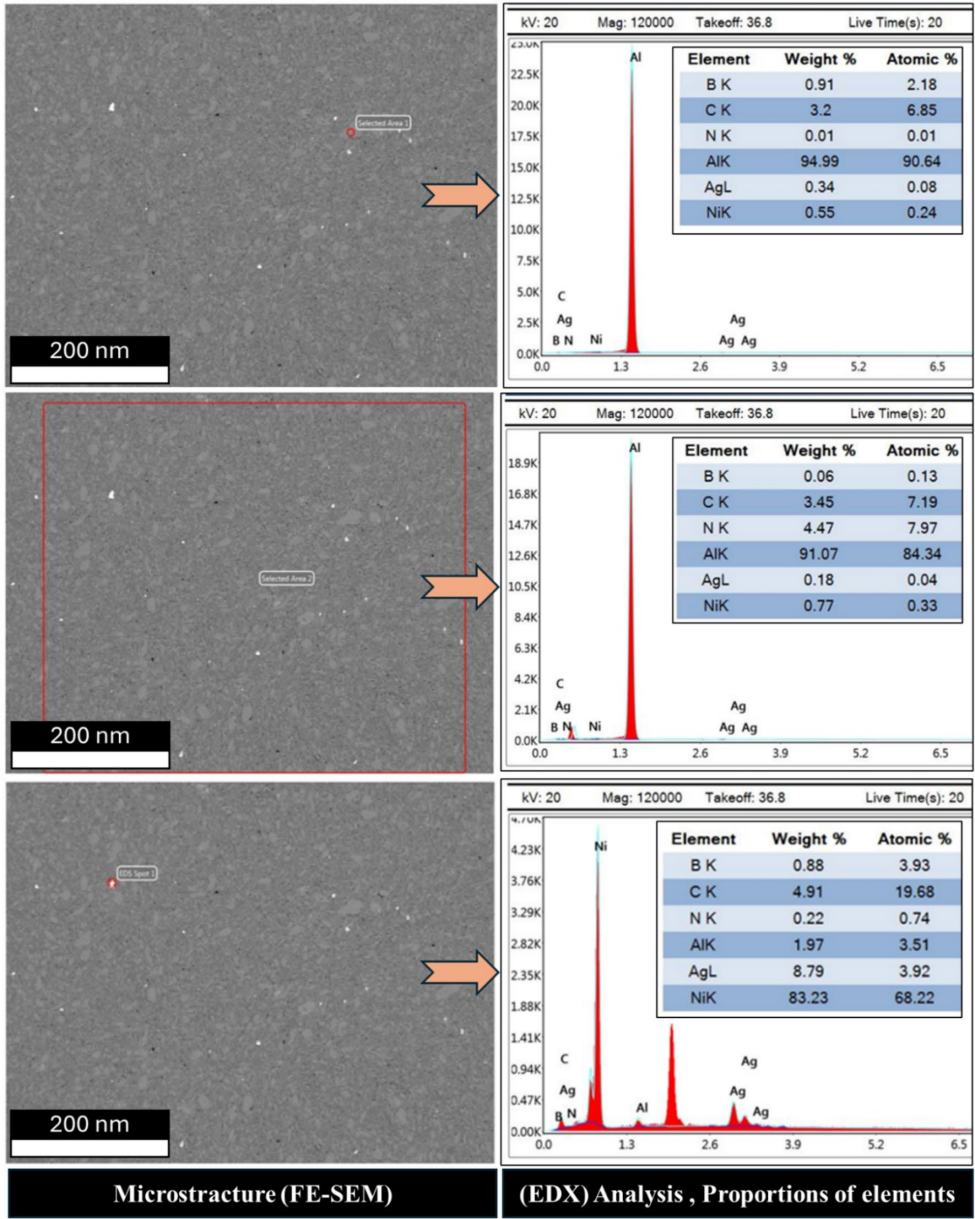
FE-SEM micrograph, EDX analysis and proportions elements of the sintered Al-0.6 wt% CNTs/h-BN nanohybrid composites reinforced at 2 wt% of nano h-BN.

**Fig. 9:**
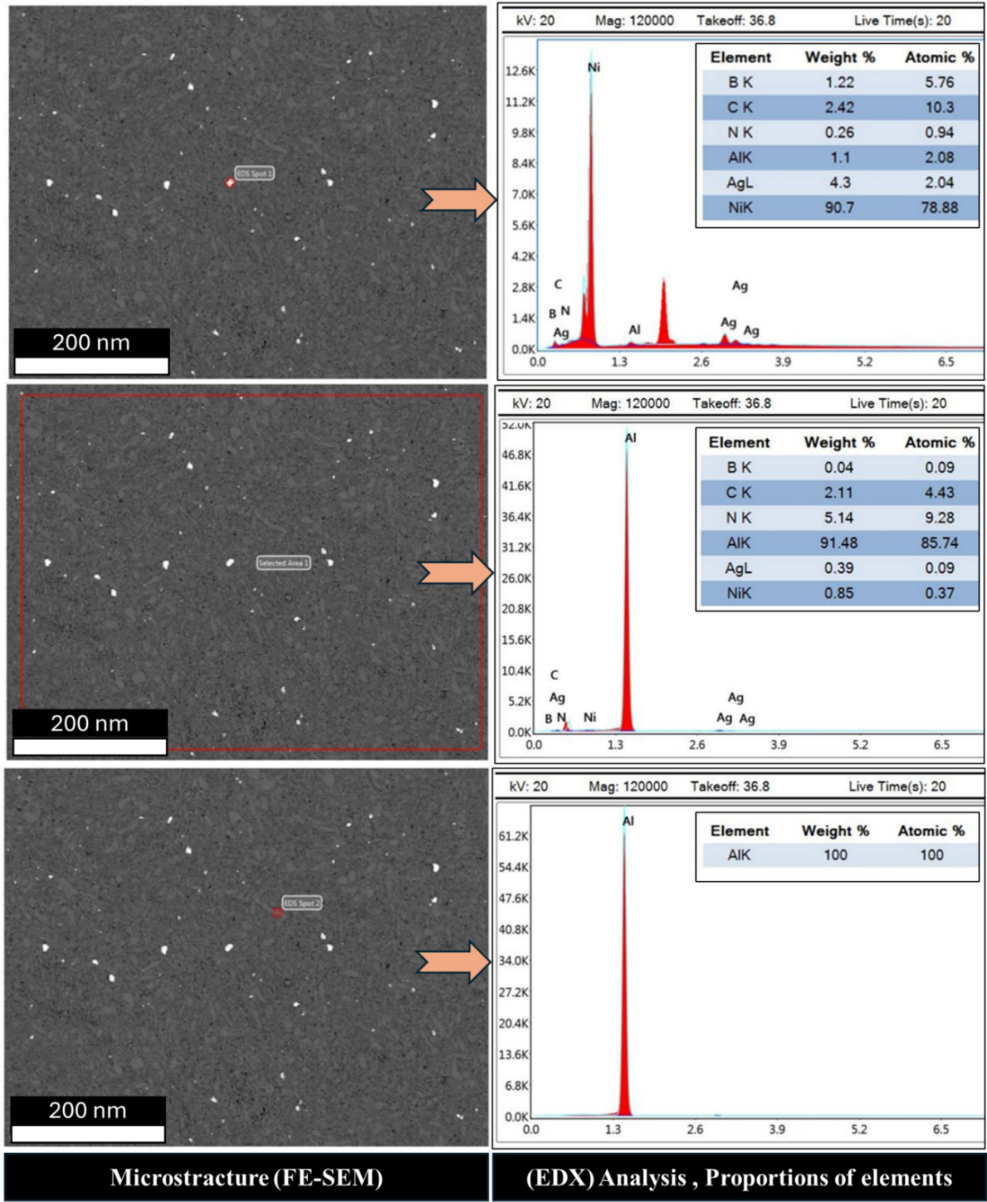
FE-SEM micrograph, EDX analysis and proportions elements of the sintered Al-0.6 wt% CNTs/h-BN nanohybrid composites reinforced at 8 wt% of nano h-BN.

**Fig. 10:**
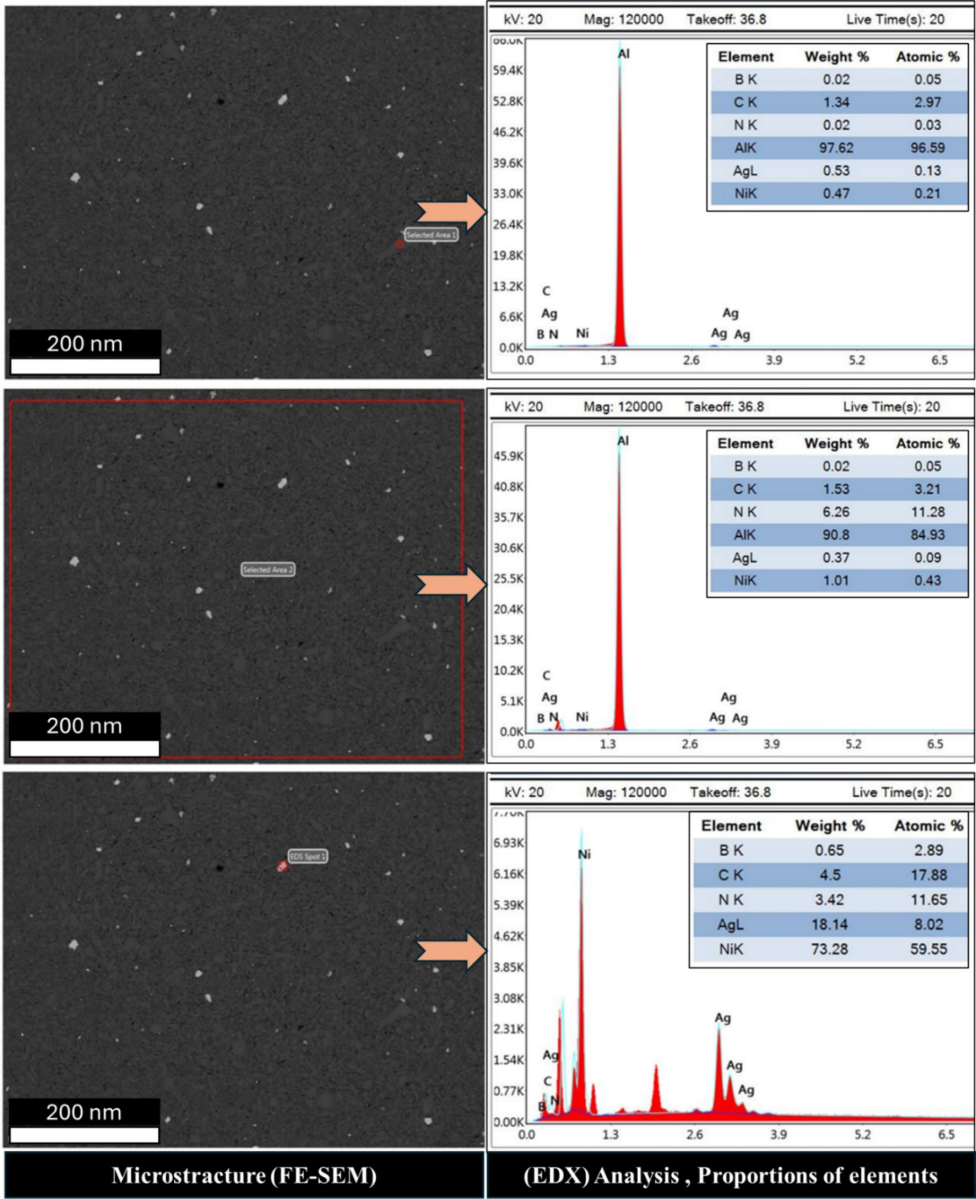
FE-SEM micrograph, EDX analysis and proportions elements of the sintered Al-0.6 wt% CNTs/h-BN nanohybrid composites reinforced at 10 wt% of nano h-BN.

To evaluate the elemental distribution within the structure, Figs. 8 and 9, and 10 illustrate the FE-SEM micrographs, EDX analyses, and elemental proportions of sintered Al-0.6 wt% CNTs/h-BN nanohybrid composites reinforced with varying amounts of h-BN. During surface scanning at 2, 8, and 10 wt% h-BN, the image on the right side of the structure displays Al grains in white-gray, while the image on the left side reveals Ni grains in white. This observation underscores the efficacy of the electroless deposition technique in coating Ag and Ni, enhancing the wettability between the Al matrix and CNTs and h-BN reinforcement, particularly evident in the case of 8 wt% h-BN. Regarding the Ni content within the entire structure, values of 0.22, 5.14, and 6.26 are observed for 2, 8, and 10 wt% h-BN, respectively. Conversely, the Ag content for the same h-BN concentrations is found to be 8.97, 0.39, and 0.37, respectively. It is noteworthy that both Ni and Ag are effectively incorporated between Al, CNTs, and h-BN. The successful prevention of agglomeration in all presented samples can be credited to the electroless deposition coating of Ni and Ag. This is particularly noticeable in the sample with 8 wt% h-BN. The findings indicate the effective integration of Ni and Ag, thereby improving the overall structural integrity and properties of the nanohybrid composite. The significance of electroless deposition is highlighted as a key factor contributing to enhanced wettability and the prevention of agglomeration. Specifically, the fig highlights that the pivotal factor driving structural changes in the nanohybrid composite is the electroless coating of h-BN particles with Ag and Ni particles. This alteration in particle structure, from a hexagonal shape with sharp edges to a spherical form, is directly associated with the electroless coating process involving Ag and Ni particles.

**Fig. 11:**
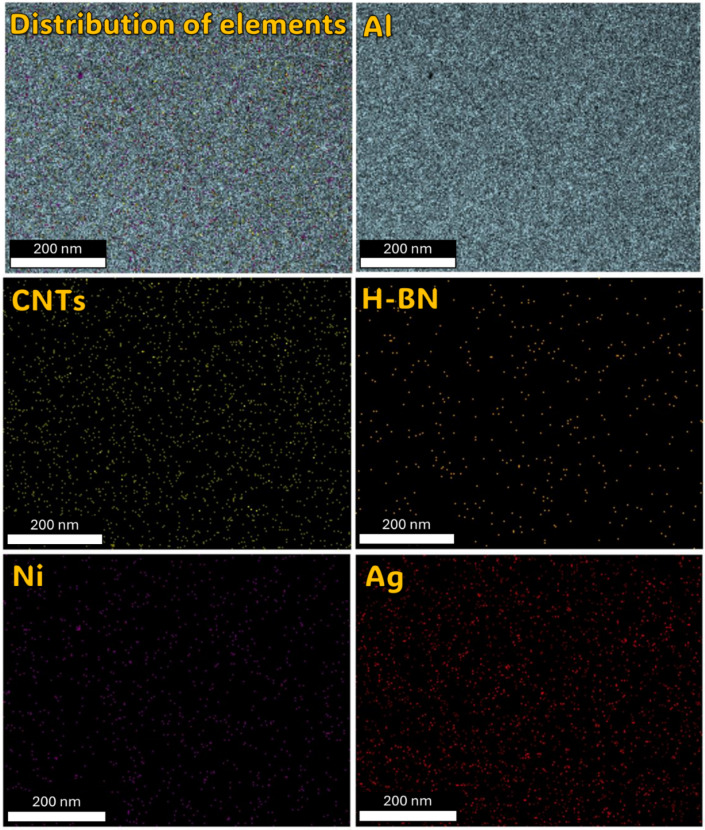
FE-SEM micrograph, Mapping analysis of the sintered Al-0.6 wt% CNTs/h-BN nanohybrid composites reinforced at 2 wt% of nano h-BN.

**Fig. 12:**
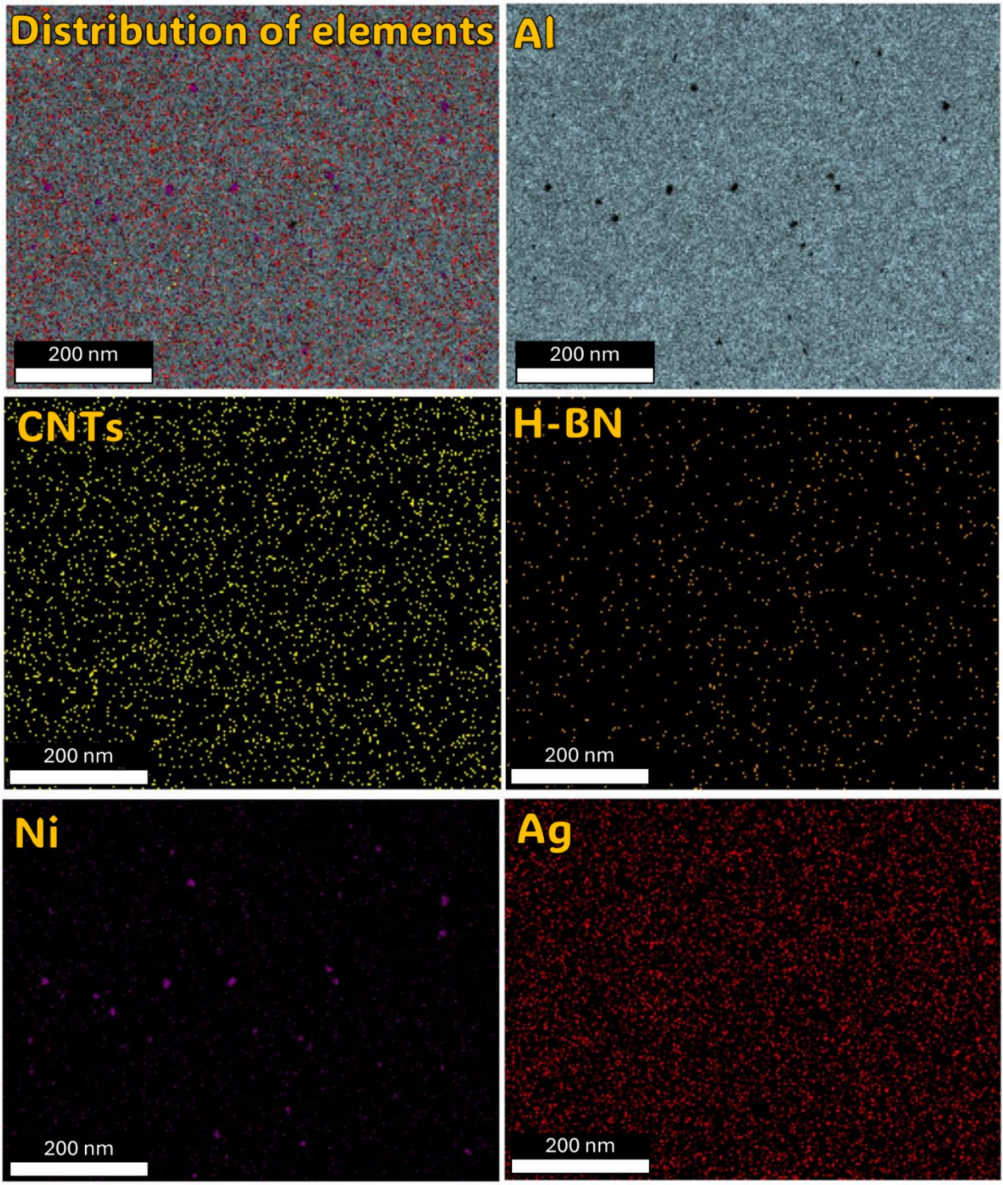
FE-SEM micrograph, Mapping analysis of the sintered Al-0.6 wt% CNTs/h-BN nanohybrid composites reinforced at 8 wt% of nano h-BN.

**Fig. 13:**
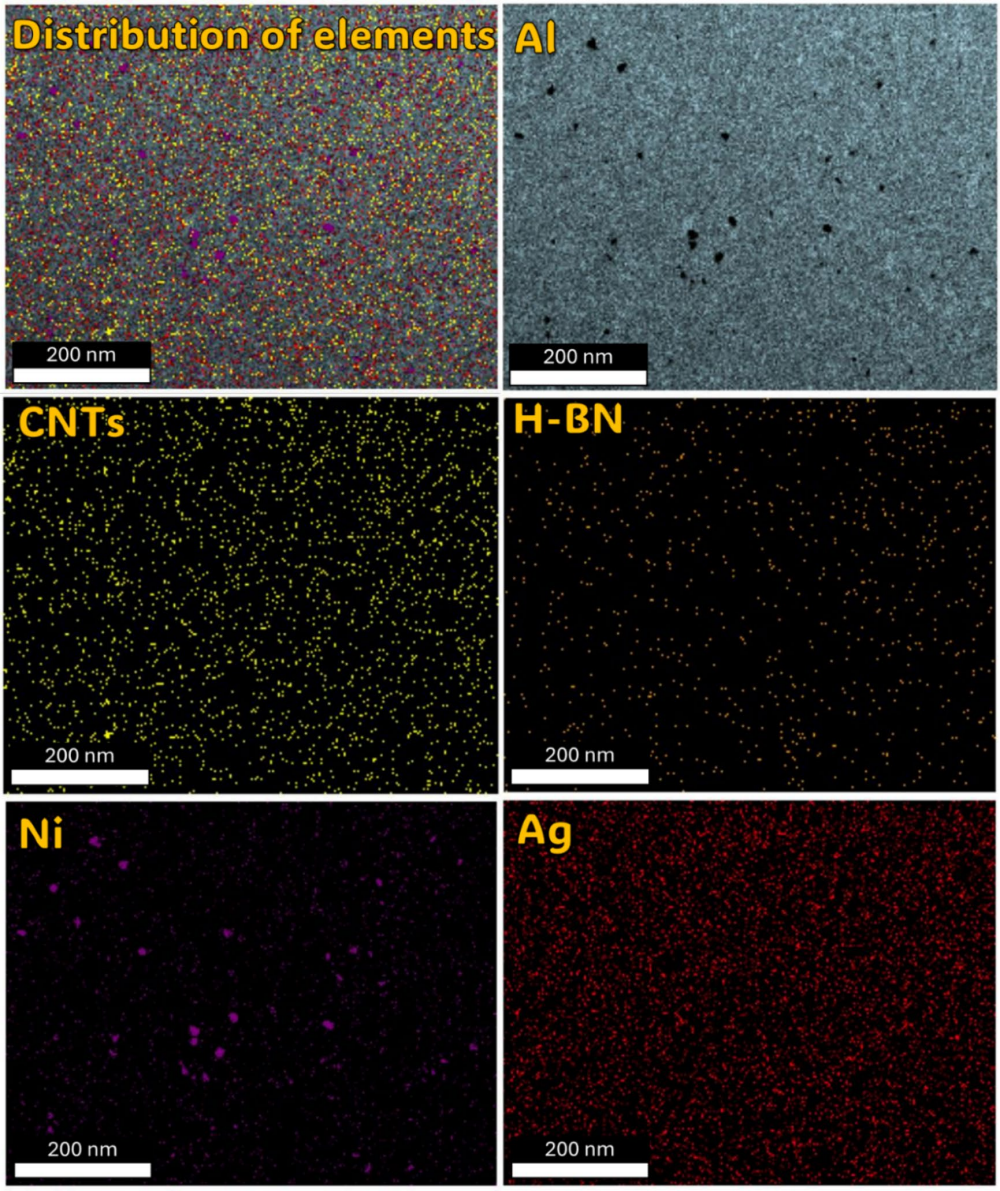
FE-SEM micrograph, Mapping analysis of the sintered Al-0.6 wt% CNTs/h-BN nanohybrid composites reinforced at 10 wt% of nano h-BN.

Understanding the role of h-BN in grain refinement opens up new possibilities for tailoring the mechanical properties of metal matrix composites. By strategically incorporating h-BN at optimal concentrations, researchers and engineers can control the grain structure and enhance mechanical performance. Thus, the investigation into the influence of h-BN content on grain refinement in metal matrix composites has revealed promising results, particularly focusing on an 2, 8, and 10 wt% h-BN composition. In the sample of Al-0.6wt.%CNTs/2wt.% h-BN, the distribution of the Al matrix is semi homogeneity, although distributions ratios of the Ag, Ni, C, and BN are very little and un-homogenous, which cause appear the voids. On the other side, in the sample of Al 0.6%CNTs/8wt.% h-BN, the scattering of Ag, Ni, C, and BN are very homogenous and suitable inside the Al matrix, which forms obstacle forward increase grains. While, in the sample of Al 0.6wt.%CNTs/10wt.% h-BN, the dispersion of Ag, Ni, C, and BN are un-homogenous which causes agglomeration of the grains inside the Al matrix. A noteworthy discovery emerges from the mapping analysis, particularly in the case of Al 0.6%CNTs/8wt.% h-BN. The application of the electroless deposition technique proves instrumental in effectively coating Ag and Ni onto h-BN and CNTs. This process fosters strong adhesion among these materials, successfully solving the problem of wettability. In the Al-0.6%CNTs/2wt.% h-BN sample, the distribution of the Al matrix is semi-homogeneous. However, the distributions of Ag, Ni, C, and BN exhibit minimal homogeneity, leading to the formation of voids. Conversely, in the Al 0.6%CNTs/8wt.% h-BN sample, the scattering of Ag, Ni, C, and BN is highly homogeneous, fitting well within the Al matrix and acting as barriers to grain growth. On the contrary, in the Al 0.6%CNTs/10wt.% h-BN sample, the dispersion of Ag, Ni, C, and BN is non-uniform, resulting in the agglomeration of grains within the Al matrix. The investigations, which involved mechanical milling and hot compaction, revealed intriguing insights into the role of h-BN in influencing grain structure and deformation behavior. During the mechanical milling and hot compaction processes, the Ag and Ni grains in the composites undergo plastic deformation. This plastic deformation necessitates harmonious deformation between adjacent grains, resulting in an increased density of dislocations within the material. The high specific surface area of carbon nanotubes (CNTs) and h-BN is found to play a crucial role in hindering the movement of dislocations and promoting dislocation pile-ups. Role of CNTs and h-BN, The microstructure analysis, as depicted in Fig. 11, illustrates that CNTs and h-BN are predominantly located at the grain boundaries. This strategic placement acts as a barrier, impeding the movement of dislocations and contributing to grain refinement. The effectiveness of this mechanism is further emphasized by the homogeneous distribution of h-BN nanoparticles throughout the matrix, as observed in the Fig. 12. Similar observations have been reported in literature for various materials, such as Al/GNPs composites, Al- Al_2_O_3_ composites, and titanium alloy with graphene nanoplatelets (Ti6Al4V GNPs)^9,34,52^. Accordingly, the strategic placement of CNTs and h-BN at grain boundaries impedes the movement of dislocations, leading to enhanced grain refinement. The homogenous distribution of h-BN throughout the matrix further highlights its effectiveness in this regard. These findings provide a foundation for developing of advanced materials with tailored mechanical properties, opening up new avenues for innovation in materials science and engineering. On the other hand, in Fig. 13. a notable increase in grain size is observed as the percentage of h-BN rises to 10%, contrasting with the 8% composition. This expansion can be ascribed to plastic deformation and the initiation of new dynamic recrystallization processes. Additionally, the concentration and agglomeration of nickel and silver grains within the surface contribute to irregularities in the microscopic structure of the material’s surface.

### Density and porosity of the Al-CNTs/h-BN hybrid composites

**Fig. 14:**
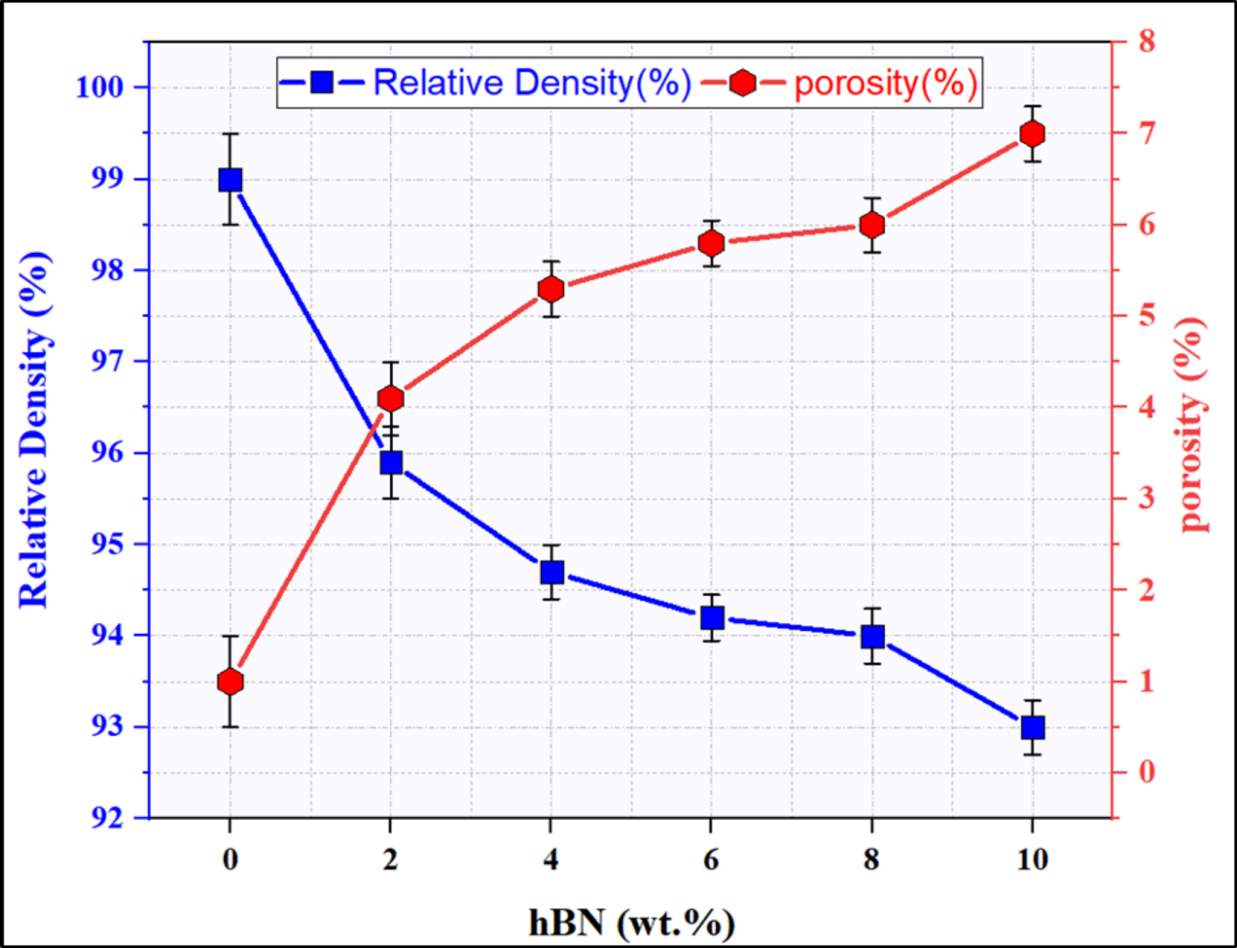
Relative density and porosity of the Al-0.6 wt% CNTs/h-BN hybrid composites reinforced with different concentrations ranging from 0 to 10 wt% h-BN.

The density of sintered Al-CNTs-based hybrid composites was measured using Archimedes’ principle, and the mixture’s role estimated their theoretical density. Figure 14 illustrates the relative density and porosity of the produced Al-0.6 CNTs hybrid nanocomposites reinforced with different ratios of 0, 2, 4, 6, 8 and 10 wt% h-BN. It shows a gradual decrease of the density with increasing the h-BN ratio Also the porosity increases, which corresponds to the decrease in relative density. It can be remarked that the relative density of the sintered Al-matrix is close to full densification, around 99%, and the relative density of the sintered hybrid composites reduced with increasing the h-BN content to around 93% for the Al-0.6 wt% CNT/10 wt% h-BN. First, the high relative density of over 93% for all produced hybrid composites is due to the self-lubricant properties of nano h-BN. During the hot-pressing process, nano h-BN acts as a solid lubricant, reducing inter-particle friction and preventing the coalescence of powder particles. This facilitates particle rearrangement and efficient packing, enabling enhanced compaction and reducing the presence of voids or porosity. Additionally, h-BN’s lubricating properties impede grain growth and agglomeration, contributing to preserving particle boundaries^15,44,48,53–55^. Also, the good distribution of CNTs and BN in the Al matrix with no agglomeration due to the coating layers of the nano Ag and Ni on their surface greatly affects the good densification Consequently, incorporating h-BN nanoparticles promotes optimal particle packing, solid-state diffusion, and inter-particle bonding, culminating in significantly improved densification. In addition, the high densifications of the sintered matrix and its hybrid composites an indication of the effectiveness of the applied production processes, such as the coating, mechanical mixing, and hot-coining process (temperature, time, and pressure) as well as the size and distribution of the ceramic particles to produce the high-density Al-0.6 wt% CNTs/h-BN hybrid nanocomposites close to the full densifications. Furthermore, the nanohybrid reinforcements had higher compressive properties compared to Al-matrix^38,53,56^.

Although the relative density of the prepared nano composites decreases by increasing the h-BN ratio, it is still very high in which the lowest value is ~ 93% for 10% h-BN which is the good densification. This can be attributed to the increase of the h-BN ratio coated with the same percentage of nano Ag and Ni, which may not be sufficient for complete capsulation of the 10% h-BN particles that causes some aggregations that form internal voids that decreases the overall density of the prepared samples. Where, reducing the relative density of nanocomposites significantly impacts their performance across mechanical, thermal, and functional domains. Higher density typically enhances mechanical strength and hardness but reducing density can enhance fracture toughness through mechanisms like crack deflection. However, density reduction may lower thermal conductivity and alter thermal expansion, affecting thermal management. It can also increase electrical resistivity and porosity, influencing material durability and suitability for applications requiring conductivity or permeability. While lighter materials benefit weight-sensitive applications, achieving density reductions may increase manufacturing costs and compromise structural integrity under stress or harsh conditions. Balancing these factors is crucial in optimizing nanocomposite performance for specific applications.

### Hardness of the Al-CNTs/h-BN hybrid composites

Hardness testing is a crucial factor in assessing the appropriateness of composites for industrial applications in sectors such as aerospace and automotive. It provides valuable insights into the composite’s ability to endure external forces without experiencing substantial plastic deformation.

**Fig. 15:**
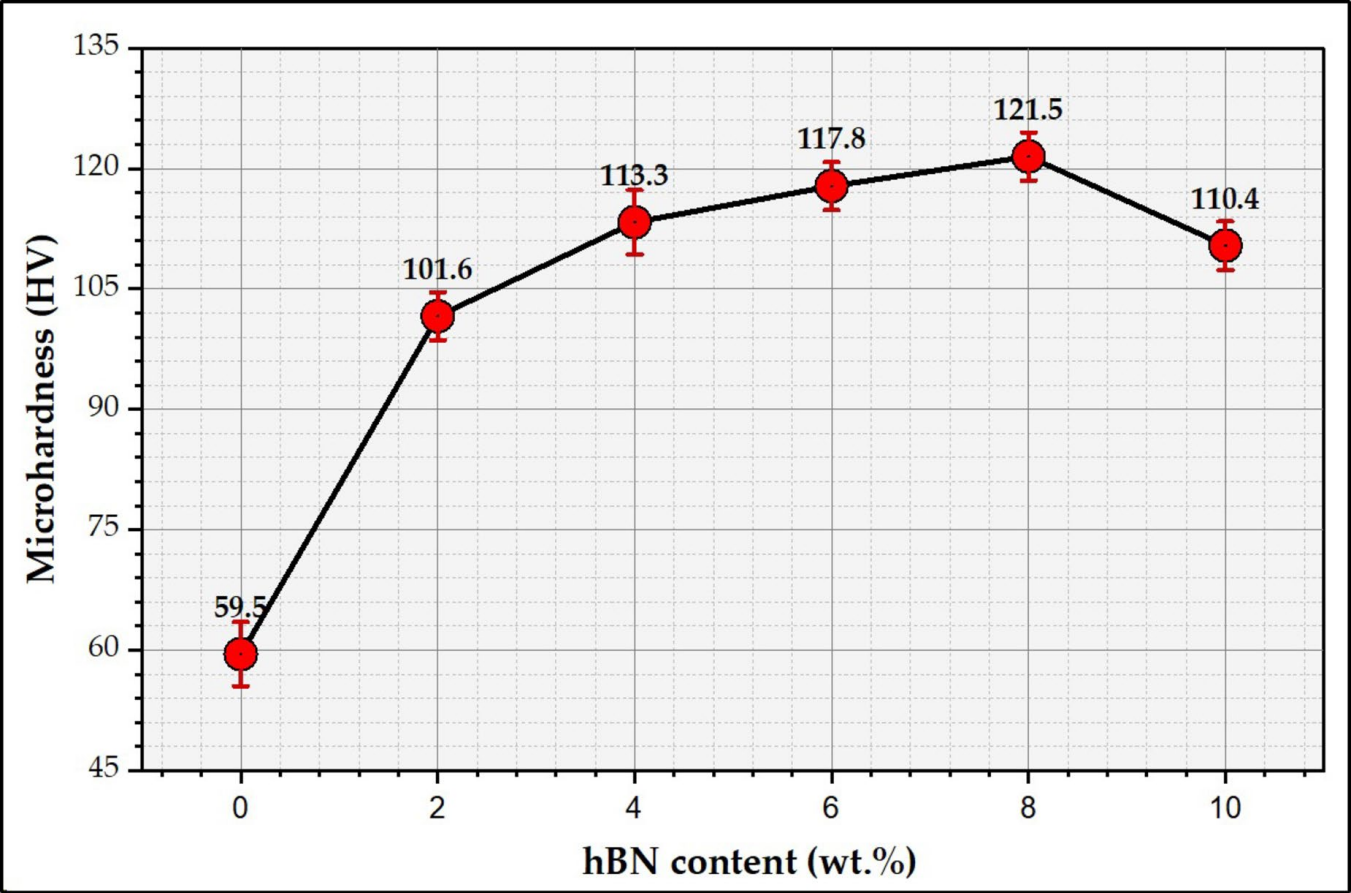
Microhardness measurements of the Al-0.6 wt% CNTs/h-BN hybrid composites reinforced with different concentrations ranging from 0 to 10 wt% h-BN.

Figure 15 represents the hardness measurements of the Al-matrix, and their composites reinforced with 0.6 wt% CNTs and different concentrations of h-BN ranging from 2 to 10 wt%. It can be commented that the hardness of the produced Al-0.6 CNTs/h-BN increased with increasing the h-BN contents except at 10 wt% h-BN, which demonstrates that the incorporation of CNTs and h-BN nano reinforcement into the Al-matrix produced using powder metallurgy enhances the hardness of the produced hybrid nanocomposites. The hardness of the sintered Al-matrix sample revealed 57 HV and increased to 121 HV for the hybrid composite reinforced with 0.6 wt% CNTs and 8 wt% h-BN. The improvement in hardness properties can be attributed to synergistic mechanisms between the distribution of the CNTs and h-BN in the matrix. The CNTs reinforce the Al-matrix by forming a strong interface with Al, efficiently transferring load and hindering dislocation movement. This results in increased hardness by impeding plastic deformation^13,21,22,43^. Simultaneously, h-BN’s lubricating properties reduce friction between particles during compaction, enabling uniform distribution of CNTs and preventing agglomeration, which can lead to improved particle dispersion^15,38,44,55^. This combination enhances the hardness of the produced hybrid composites, combining CNTs’ strengthening effect and h-BN’s facilitation of homogeneous dispersion, collectively advancing the mechanical performance of the aluminum matrix. Furthermore, the combination of h-BN in the matrix can also influence the sintering behavior of the Al-CNTs/h-BN hybrid composite; the presence of h-BN can aid in preventing excessive grain growth during sintering, maintaining a refined microstructure that can lead to increased hardness. Due to the combination effect of the addition of 0.6 CNTs with the h-BN concentrations of 2, 4, 6, 8 and 10 wt%, the hardness of the produced composites improved over the Al-matrix sample by around 42, 91, 98, 105 and 85%, respectively. In addition, it can be concluded that the hardness measurements of the Al-matrix and their hybrid composites are compatible with the near-full densification properties for the produced hybrid nanocomposites Fig. 7. The presence of a ceramic material such as h-BN with its high hardness and good distribution with a good adhesion with the Al matrix particles helps in improving the total hardness of the final prepared nano composites. Also, h-BN acts as an internal ball that increases the refining of the Al particles consequently grain refinement takes place and so hardness increases according to Hall-Petch equations^53,54,57–59^.

### Compressive strength

**Fig. 16:**
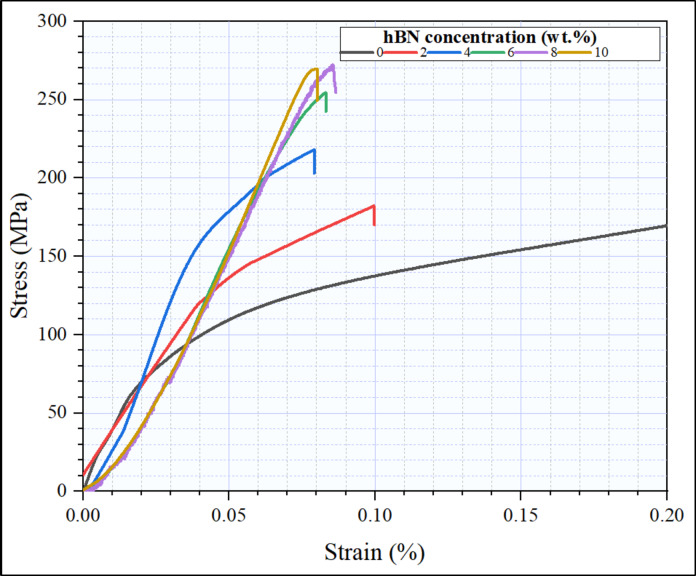
Stress strain curve of the Al-0.6 wt% CNTs/h-BN hybrid composites reinforced with different concentrations ranging from 0 to 10 wt% h-BN.

The comprehensive analysis of the compressive strength values for the investigated composites, meticulously presented in Fig. 16 indicates a discernible enhancement in compressive strength as the h-BN content increases, with the exception being the sample involving 10 wt% h-BN. Simultaneously, the strain at which failure transpired in the sintered specimens manifests an inverse correlation with the escalating h-BN content except in the sample at 10wt.% h-BN, as clearly illustrated in Fig. 16. To provide additional clarity, the corresponding strain values for h-BN additions of 2, 4, 6, 8, and 10 wt% were measured at 9.95, 7.9, 8.35, 8.53 and 8.00% respectively as shown as Table 3. The highest compressive strength (272.3 MPa) was identified at h-BN content of 8 wt%, a phenomenon attributed to a combination of factors. These include the effective dispersion of h-BN within the aluminum matrix, which optimizes interfacial interactions and enhances structural integrity. Additionally, at this specific concentration, the detrimental effects associated with excessive h-BN agglomeration and porosity are mitigated, contributing to the observed optimal compressive strength. The diminished compressive strength observed in the sintered specimens with a 10 wt% h-BN content can be attributed to a confluence of factors, including porosity, the agglomeration of h-BN within the aluminum matrix, the degree of anisotropy exhibited by h-BN grains in a specified direction, the multi-dimensional spatial distribution of these components, and the resultant imposition of multi-axial stresses, as elaborated in reference^44,45,48,55,60^.

**Table 3:** The following table shows the compressive strength and strain values for all sintered samples.

h-BN (wt%)	Compressive strength (MPa)	Strain (%)
0%	169.3	0.2
2%	188.6	0.099
4%	258.53	0.079
6%	220.6	0.083
8%	272.35	0.085
10%	271.9	0.08

### Tribological of the Al-CNTs/h-BN hybrid nanocomposites

Numerous parameters play a crucial role in influencing the performance of friction composite materials intended for ball bearing applications. These include tribological characteristics such as sliding speed, normal load, and frictional force. Additionally, factors related to additives, such as the type of reinforcement, dispersion of reinforcements, coating mechanism, and bonding between the reinforcement and matrix, are also significant contributors. To elucidate the influence of h-BN inside Al-0.6wt.%CNTs on the wear rate of hot compacted samples, a comprehensive understanding of the crystal structure of CNTs and h-BN is essential^13,31,38,44,61,62^.

The tribological characteristics of CNT-reinforced composites can be impacted by structural characteristics, including: (i) the strong metallurgical bond formed through the Interconnected covalent bonds between carbon atoms with CNTs, leading to an increased Density of dislocations in the composite; (ii)The singular and extensive molecular structure of carbon nanotubes, characterized by the absence of grain boundaries compared to other metallics materials. Furthermore, the crystal lattice of h-BN nitride is characterized by hexagonal rings forming thin, parallel planes. Boron (B) and nitrogen (N) atoms are covalently bonded within each plane, forming angles of 120 degrees among two layers. This bonding pattern involves each boron atom being bonded to three nitrogen atoms and vice versa. The interconnection of these planes is facilitated by weak Van der Waals forces. The B-N bond exhibits partial ionic character, resulting in cleavage faces with both positive and negative charges, enhancing surface energy and increasing interface friction. The localization of electrons in the pz nonhybridized orbital, attracted by highly electronegative nitrogen atoms, contributes to the distinct features of h-BN^15,39,54^. The interlayer distance of h-BN is measured at 3.33 Å^39,45,54^. resulting in a compact crystalline structure with strong interlayer bonding. This enhanced bonding, particularly between layers, can lead to varied lubrication effects on Cu-based composites. The layered structure, characterized by weak Van der Waals bonding, facilitates sliding movement of parallel planes, making it easier to shear along the basal plane of the crystal structure. While weak bonding between planes provides low shear strength in the direction of sliding movement, it imparts high compression strength perpendicular to the sliding direction. Consequently, the crystal structure of h-BN is a pivotal parameter influencing wear in the specimens. Additionally, the presence of unreacted h-BN in the grain boundaries of the matrix particulate is another contributing factor to the observed increase in wear rate.

#### **Wear of the Al-CNTs/h-BN** hybrid nanocomposites

**Fig. 17:**
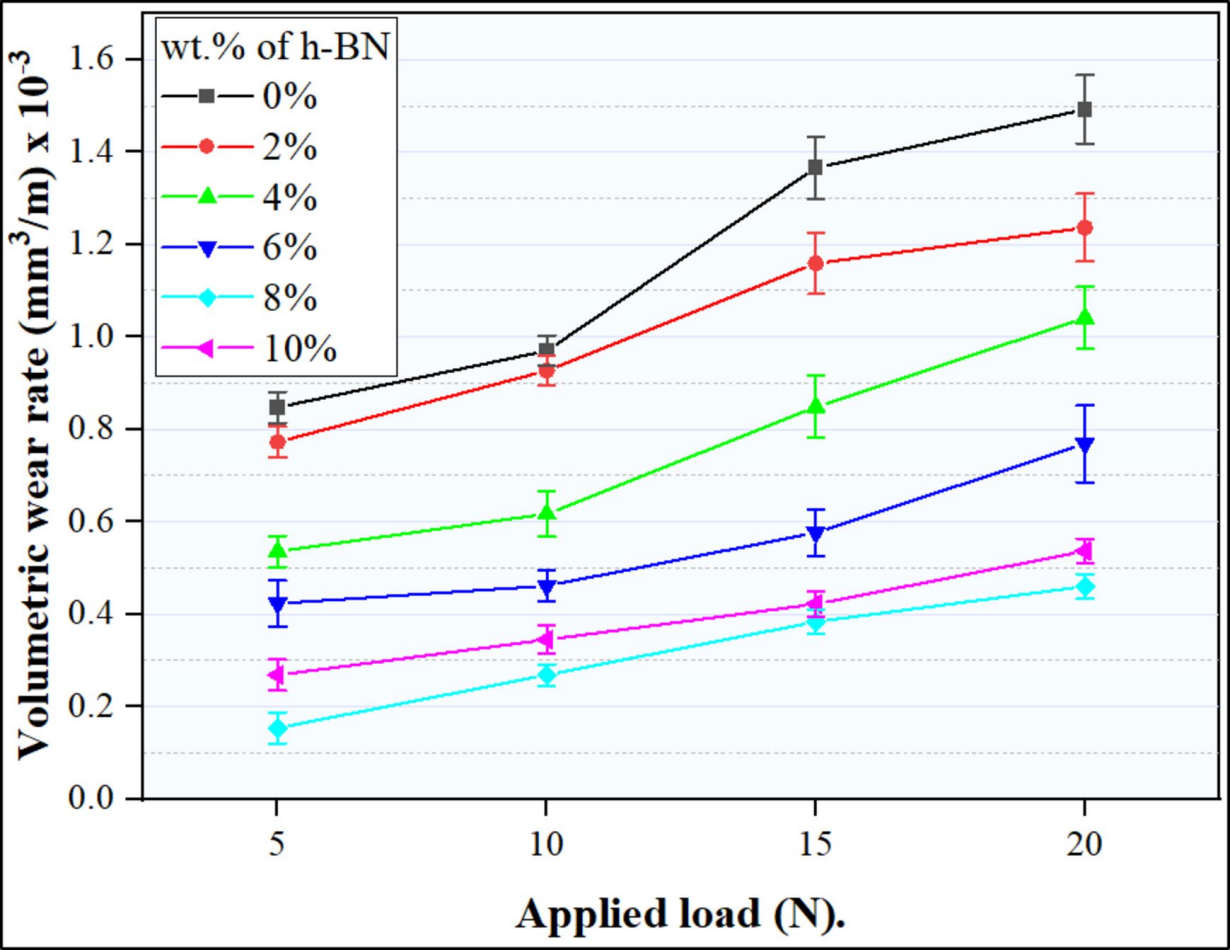
Volumetric wear rate of Al- 0.6 wt% CNTs/h-BN composites tested with several loads at kept sliding speed of 1.5 m/s.

Figure 17 illustrates the variation in wear rates between Al matrix and Al-CNTs/h-BN nanocomposites under dry sliding wear conditions at various loads, maintaining a constant sliding velocity of 1.5 m/s. The observation reveals a consistent rise in wear rates as the applied load increases across all tested samples. This indicates a direct correlation between wear penetration depth of wear in the consolidated specimens and the loads in the wear tests. Elevating the load leads to an increased penetration depth of the indenter, consequently causing a higher wear rate. A.M.I. abu-oqail et al.^13,48^. Also, present analogous findings, emphasizing that the load’s impact on the specimen mirrors the indentation response. In line with this observation, the obtained results align with Archard’s law, stating that the rate of wear increases directly with the applied load, yet decreases inversely with the hardness of the softer material in the mating pair. A novel finding reveals that the wear rate of the sample reinforced with 8wt.% h-BN within Al-0.6wt.%CNTs remains the lowest under varying applied loads. This leads to the conclusion that this case represents an optimum; This is due to the h-BN’s more robust bonding interlayer, leading to potential differences in lubrication effects on Al-based composites. Furthermore, based on the information provided in Fig. 10, it is evident that the wear rate shows increasing in the case of Al-0.6%CNTs-0%h-BN unreinforced sample. While the wear rate presents a decreasing pattern as the concentration of hexagonal boron nitride (h-BN) rises within the Al-0.6%CNTs composite samples. This inverse correlation is observable in the Al-0.6%CNTs-2%h-BN and Al-0.6%CNTs-4%h-BN specimens, where the wear rate decreases with the successive increase in h-BN content. Conversely, in the case of the Al-0.6%CNTs-6%h-BN, and Al-0.6%CNTs-10%h-BN, samples, there is an increase in the wear rate. A significant observation pertains to the altered behavior witnessed in the Al-0.6%CNTs-8%h-BN sample, where the wear rate remains relatively constant. This finding indicates that, unlike the other specimens with varying concentrations of hexagonal boron nitride (h-BN), the introduction of 8% h-BN in the Al-0.6%CNTs composite, does result in a noticeable stability and decrease in the wear rate. The stability in the wear rate in this composition suggests a unique interaction or threshold effect between the constituents, highlighting the need for further investigation to comprehend the underlying mechanisms governing wear resistance in this specific configuration. The primary explanation for these behaviors lies in the facilitation of hexagonal boron nitride (h-BN) through its layered structure connected by relatively weak bonds, such as Van der Waals forces. This structural arrangement allows for sliding movement between parallel planes and facilitates easier shearing along the basal plane of the crystal structure. The resultant weak bonding among planes imparts low shear strength in the sliding direction while contributing to high compression strength perpendicular to the sliding direction. Consequently, the crystal structure of h-BN emerges as a pivotal factor influencing the wear characteristics of the specimens. On the other hand, the incorporation of carbon nanotubes (CNTs) and h-BN nano powders serves to fortify the aluminum matrix, increasing its hardness and thereby enhancing wear resistance. Additionally, the adhesion between the reinforcement and the matrix plays a crucial role in influencing the wear resistance of the specimens.

#### COF of the Al-CNTs/h-BN hybrid composites

**Fig. 18:**
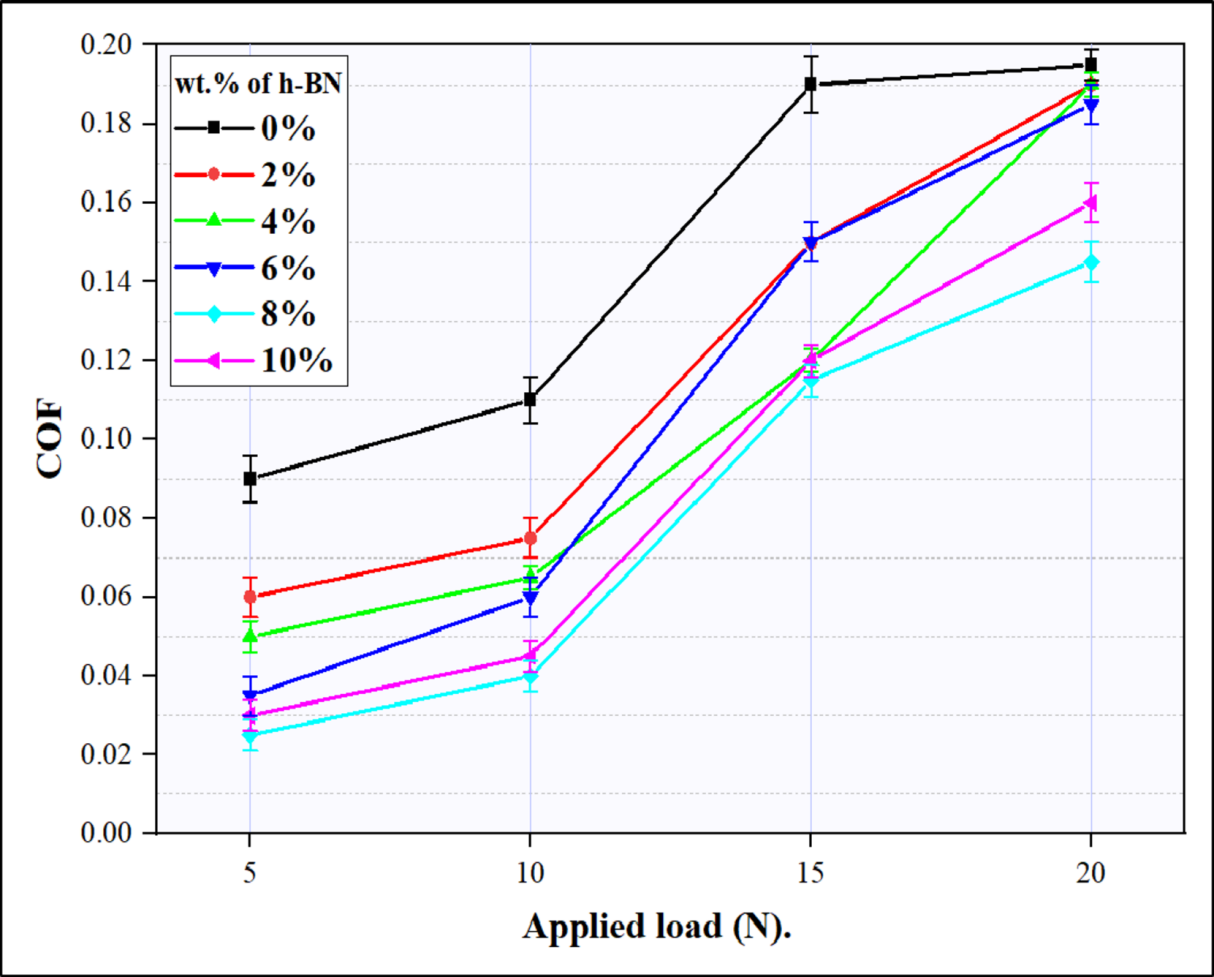
COF of Al- 0.6 wt% CNTs/h-BN composites tested with several loads at kept sliding speed of 1.5 m/s.

Figure 18 illustrates the changes in the coefficient of friction concerning the normal load under a consistent sliding speed of 1.5 m/s for both the Al-0.6wt.% CNTs/0wt.% h-BN and Al-0.6wt.% CNTs/h-BN nanocomposites. The increasing applied load increases the friction coefficient for all the composites synthesized. However, friction coefficient decreased with the increasing h-BN content in the composites. a new hint can be observed that, the lowest COF was obtained at the case ofAl-0.6wt.% CNTs/8wt.% h-BN. this is due to the unique dispersion of h-BN in Al-CNTs composites. This observation implies that the incorporation of h-BN into the composite material leads to improved wear resistance. The rationale behind this improvement can be explained by the inherent properties of the reinforcement particles, specifically the harder nature of h-BN. In such composites, the role of harder reinforcement particles is crucial in withstanding the applied load during wear conditions. These particles act as asperities, which are small, elevated features on the surface, and play a significant role in tribological interactions. As the harder reinforcement particles, such as h-BN, withstand the load, they act as barriers that reduce the contact area between the pin and counter face during sliding or rubbing. This reduction in contact area has a direct impact on the friction coefficient of the material. A lower friction coefficient is indicative of reduced resistance to motion between the surfaces in contact. Therefore, the introduction of h-BN in the Al-CNTs matrix contributes to the reduction of friction coefficient, leading to enhanced wear resistance in the Al-CNTs/h-BN composites. This observation aligns with established principles in material science and tribology, where the choice and incorporation of appropriate reinforcement materials can significantly influence the mechanical and tribological properties of composite materials. In the context of Al-CNTs/h-BN composites, the harder h-BN particles play a critical role in improving wear resistance by minimizing pin-counter face contact area and subsequently reducing friction. Finally, the overall improvement in wear resistance and COF observed in Al-CNTs/h-BN nanocomposites can be attributed to the synergistic influence of the unique properties of carbon nanotubes (CNTs) and hexagonal boron nitride (h-BN). This combination contributes to an overall enhancement in wear resistance, highlighting the efficacy of the composite materials in strengthening the aluminum matrix and augmenting its resistance to wear.

**Fig. 19:**
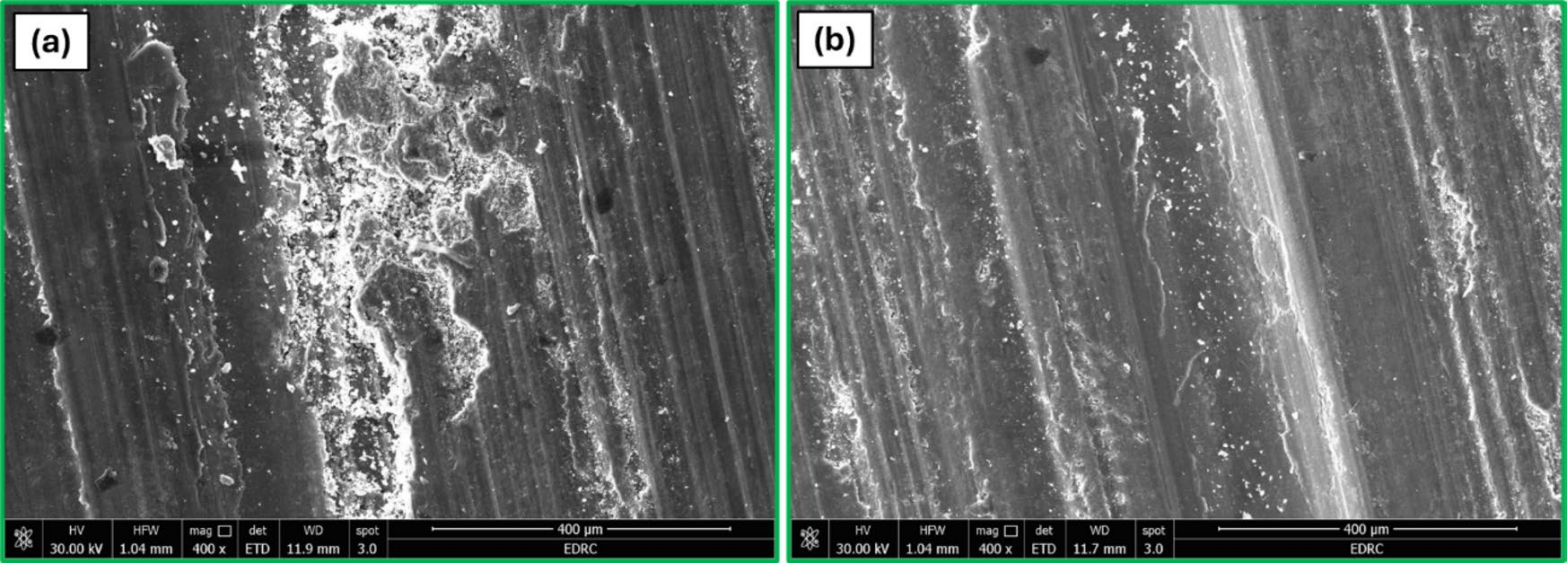
(**a**) Worn surfaces SEM images of the sintered Al-0.6 wt% CNTs/h-BN nanohybrid composite samples with varying amounts of h-BN reinforcement of (**a**) 0 wt%, (**b**) 8 wt%.

Figure 19 shows the SEM images of the worn surfaces for the Al-0.6 wt% CNTs/h-BN nanohybrid composite samples with varying amounts of h-BN reinforcement. For the sample with 0 wt% h-BN (pure Al-CNT composite), the worn surface exhibits significant plastic deformation and material removal. Deep grooves and flowing marks are evident, indicating abrasive wear as the dominant mechanism. The surface appears rough and uneven, suggesting that the material experiences severe wear under the applied conditions, indicative of abrasive wear, which aligns with the higher COF and wear rate observed in Figs. 17 and 18. This behaviour is typical for soft aluminium alloys without sufficient reinforcement to enhance their wear resistance. In contrast, the sample with 8 wt% h-BN shows a markedly different wear surface morphology. The worn surface appears smoother and more uniform, with fewer deep grooves and less evident plastic deformation. This suggests a transition to a milder wear regime, likely due to the lubricating effect of h-BN particles. The presence of h-BN appears to reduce direct metal-to-metal contact, thereby minimizing adhesive wear and material transfer. The 8 wt% h-BN sample also exhibits some fine, shallow grooves and small debris particles on the surface. These features indicate a combination of mild abrasive wear and the formation of a protective tribofilm. This smoother surface corresponds to the lower COF and reduced wear rate seen in Figs. 17 and 18 for the 8 wt% h-BN sample across various loads. The h-BN particles likely act as solid lubricants, forming a thin layer on the wear surface that reduces friction and protects the underlying material from severe wear. Furthermore, the 8 wt% h-BN sample shows evidence of delamination in some areas, characterized by the presence of flake-like debris and small pits on the surface. This delamination wear mechanism is often observed in composite materials and can be attributed to subsurface crack propagation parallel to the sliding direction, eventually leading to the removal of thin layers of material. The improved wear resistance of the 8 wt% h-BN sample can be attributed to several factors. First, the h-BN particles act as load-bearing elements, distributing the applied stress more evenly across the material. Second, the lubricating properties of h-BN reduce friction between the sliding surfaces. Third, the presence of h-BN may enhance the overall hardness and strength of the composite, making it more resistant to plastic deformation and material removal.

**Fig. 20:**
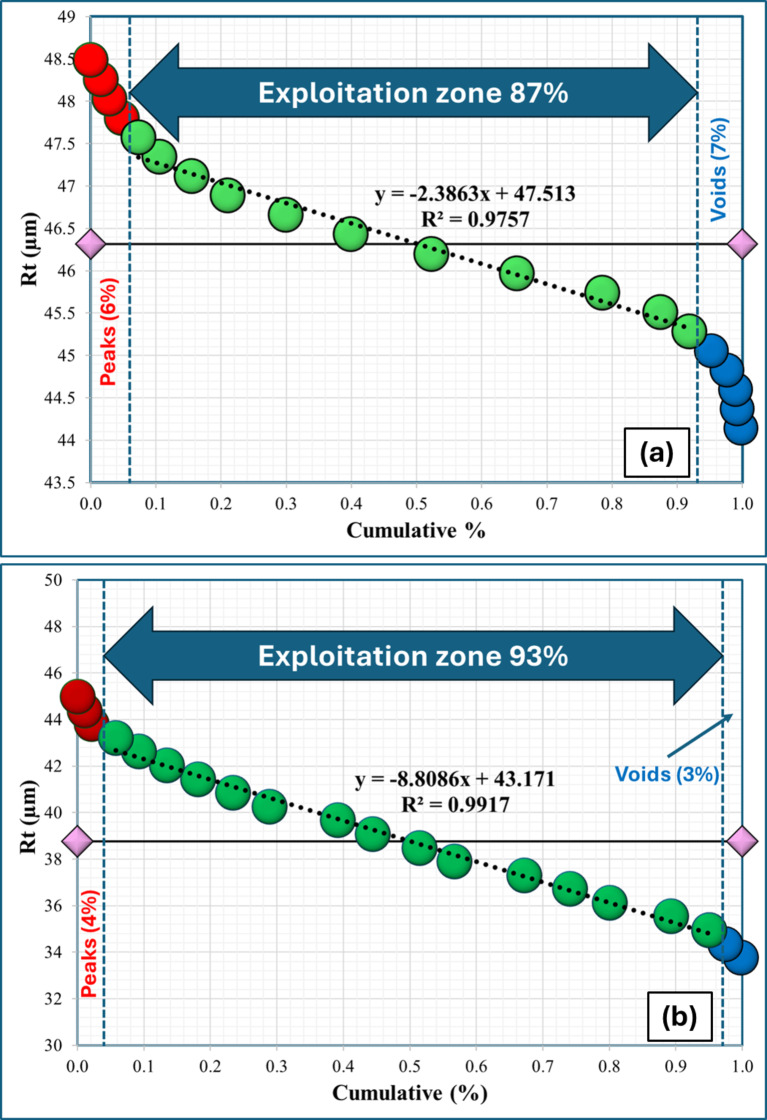
Abbott-Firestone analysis of the sintered Al-0.6 wt% CNTs/h-BN nanohybrid composite samples with varying amounts of h-BN reinforcement of (**a**) 0 wt%, (**b**) 8 wt%.

Figure 20 shows the surface topography and wear characteristics of the Al-0.6 wt% CNTs/h-BN Which was carried out at higher lode of 20 N. These analyses significantly enhance our understanding of the tribological behavior of the composites, particularly for the pure sample (0% h-BN) and the sample with 8% h-BN content, which exhibited the best mechanical properties. Figure 20 presents the Abbott-Firestone curves for both samples, offering a quantitative representation of the surface height distribution. These curves are instrumental in characterizing the bearing area and fluid retention properties of the worn surfaces. The pure Al sample (0% h-BN) exhibits a steeper curve with a peak area and a voids area, indicating a more irregular surface topography. In contrast, the 8% h-BN sample shows a flatter curve with reduced peak area and voids area values, suggesting a smoother, more uniform wear surface. The exploitation zone (93%) for the 8% h-BN sample is significantly higher than that of the pure sample (87%), indicating a more wear-resistant surface. The reduced peak and voids values for the 8% h-BN sample corroborate the SEM observations of a smoother wear track (Fig. 19). These parameters suggest that the 8% h-BN sample exhibits better load-bearing capacity and reduced friction. These findings align well with the previously discussed SEM images (Fig. 19) of the worn surfaces and the tribological test results (Figs. 17 and 18). The smoother surface and more favorable Abbott-Firestone curve parameters of the 8% h-BN sample explain the observed improvements in wear rate and coefficient of friction. The presence of h-BN likely contributes to the formation of a more uniform tribofilm, which protects the underlying material and promotes a milder wear regime.

## Conclusions

A new attempt was successfully made for the first time through adding hexagonal boron nitride (h-BN) into Aluminum-Carbon nanotubes (Al-0.6 wt% CNTs) nanocomposites by an innovative coated with silver (Ag) and nickel (Ni) using a novel electroless chemical deposition technique. In order to improve the wettability and dispersion between reinforcement and matrix, and solve many difficulties occur in ball bearings such as: (i) increase temperature, (ii) high pressure, (iii) wear rate, and(iv) coefficient of friction between contacting parts of the bearings. Thus, different h-BN contents as 2, 4, 6, 8 and 10 wt% are mixed and sintered by high-energy ball milling and hot compaction process. Various analyses and tests were used by microstructure, physical, mechanical, and tribological in order to explore the new characteristics and properties. The conclusions drawn from the obtained results and observations in this work can be summarized as follows:

1. The increase in h-BN content from 0 wt% up to 10 wt% resulted in a significant reduction in relative density from 99% up to 93%. This decline in relative density was primarily due to the formation of micropores during the consolidation process. Achieving a homogeneous dispersion of coated h-BN nanoparticles within the Al matrix was successful up to a concentration of 8 wt% However, when the h-BN content was further increased to 10 wt%, notable particle agglomeration occurred.
2. The inclusion of a hybrid composite, reinforced with 0.6 wt% CNTs and 8 wt% h-BN, resulted in an increase in hardness from 57 HV in the sintered Al-matrix sample to 121 HV. This improvement in hardness can be attributed to synergistic mechanisms stemming from the efficient distribution of both CNTs and h-BN within the matrix.
3. The highest compressive strength (272 MPa) was identified at an h-BN content of 8 wt%. This optimal strength is attributable to the synergistic effects of a well-balanced dispersion of h-BN within the aluminum matrix, fostering improved interfacial interactions and structural robustness. At this concentration, potential drawbacks associated with excessive h-BN agglomeration and porosity are effectively mitigated, thus culminating in the attainment of the highest observed compressive strength.
4. With increasing the content of h-BN from 0% up to 8% h-BN inside the matrix of Al-0.6% CNTs, the wear rate decreased from value of 1.49 × 10 − 3 mm3/m at 0% h-BN to value 0.46 × 10 − 3 mm3/m at 8% h-BN nanocomposite at 20 N load. Conversely, the wear rate increased for the sample containing 10% h-BN, attributable to the decline in hardness and relative density resulting from the presence of h-BN aggregates, as observed in the microstructure. At the same time, the COF occurred same behavior of wear rate. Specifically, the coefficient of friction decreased at 20 N load from 0.195 for the Al-0.6% CNTs-0% h-BN composite to 0.145 for the Al-0.6 wt% CNTs/8 wt% h-BN nanocomposite.

Finally, the Al-0.6 wt% CNTs/8 wt% h-BN nanocomposite displayed exceptional and distinctive properties, providing a testament to the efficacy of this innovative approach in augmenting the material’s inherent characteristics. The next steps in the future work related to nanocomposites involve several avenues to enhance their properties and applications. These include optimizing synthesis methods for better uniformity and distribution of nanoparticles, exploring different nanoparticles (e.g., metal oxides, carbon nanotubes, graphene) to achieve property enhancements, and functionalizing nanoparticles to improve compatibility with the matrix and impart specific properties like antimicrobial activity, electrical conductivity, or thermal stability. Additionally, conducting detailed testing to understand nanocomposite limits, developing computational models to predict behavior, and tailoring nanocomposites for specific applications (e.g., aerospace, automotive, biomedical, electronics) are crucial. Evaluating environmental and health impacts and partnering with industry and academic institutions for collaborative research will further enhance nanocomposite properties and expand their applications.

## Data Availability

The datasets used and/or analyzed during the current study are available from the corresponding author on reasonable request.
